# Discovery and characterization of noncanonical E2-conjugating enzymes

**DOI:** 10.1126/sciadv.adh0123

**Published:** 2024-03-27

**Authors:** Syed Arif Abdul Rehman, Chiara Cazzaniga, Elena Di Nisio, Odetta Antico, Axel Knebel, Clare Johnson, Alp T. Şahin, Peter E. G. F. Ibrahim, Frederic Lamoliatte, Rodolfo Negri, Miratul M K Muqit, Virginia De Cesare

**Affiliations:** ^1^ MRC Protein Phosphorylation and Ubiquitylation Unit, Sir James Black Centre, School of Life Sciences, University of Dundee, Dow Street, Dundee DD1 5EH, Scotland, UK.; ^2^ MRCPPU Reagents and Services, School of Life Sciences, University of Dundee, Dow Street, Dundee DD1 5EH, Scotland, UK.; ^3^ Department of Biology and Biotechnologies “C. Darwin”, Sapienza University of Rome, via dei Sardi, 70 00185 Rome, Italy.; ^4^ Computational Biology, School of Life Sciences, University of Dundee, Dundee, UK.; ^5^ Drug Discovery Unit, Division of Biological Chemistry and Drug Discovery, University of Dundee, Dow St, Dundee DD1 5EH, UK.; ^6^ Institute of Molecular Biology and Pathology, CNR, Via degli Apuli 4, 00185 Rome, Italy.

## Abstract

E2-conjugating enzymes (E2s) play a central role in the enzymatic cascade that leads to the attachment of ubiquitin to a substrate. This process, termed ubiquitylation, is required to maintain cellular homeostasis and affects almost all cellular process. By interacting with multiple E3 ligases, E2s dictate the ubiquitylation landscape within the cell. Since its discovery, ubiquitylation has been regarded as a posttranslational modification that specifically targets lysine side chains (canonical ubiquitylation). We used Matrix-Assisted Laser Desorption/Ionization-Time Of Flight Mass Spectrometry to identify and characterize a family of E2s that are instead able to conjugate ubiquitin to serine and/or threonine. We used structural modeling and prediction tools to identify the key activity determinants that these E2s use to interact with ubiquitin as well as their substrates. Our results unveil the missing E2s necessary for noncanonical ubiquitylation, underscoring the adaptability and versatility of ubiquitin modifications.

## Teaser

Spotlight on noncanonical E2s: UBE2Qs target nonlysine residues via a YHW triad independently of E3 ligases.

## INTRODUCTION

Attachment of one or more ubiquitin molecules to a substrate requires the sequential activity of an E1-activating enzyme (E1), an E2-conjugating enzyme (E2s), and an E3 ligase (E3). This process, named ubiquitylation (or ubiquitination), plays a major role in various pathways during cell life and death, including but not limited to cell division and differentiation, response to environmental stress, immune response, DNA repair, and apoptosis (*1*–*6*). The human genome encodes around 40 E2s (*7*) and more than 700 E3 (*8*, *9*). E3s ligases are divided into subfamilies depending on the presence of either a RING (really interesting new gene) or an HECT (homologous to the E6AP carboxyl terminus) domain (*10*). RING E3 ligases represent the vast majority of known E3s (*8*), and they represent essential activators that facilitate the direct transfer of ubiquitin from the E2s to the substrate by decreasing the *K*_m_ and increasing *K*_cat_ for both their substrates: Ub-loaded E2 and the protein to be modified. Besides the activating role of the RING E3s, E2s possess key activity determinants that direct the transfer of ubiquitin to the substrate and govern both the type of ubiquitin linkage and the extent of ubiquitin modification (*11*, *12*). E2s that functionally interact with RING E3 ligases have intrinsic reactivity toward lysine, the canonical ubiquitylation target. However, other hydroxyl-containing amino acid and biomolecules, such as serine, threonine, sugars, and the bacterial liposaccharide, have also been found to be targeted by ubiquitin (*13*–*23*) and the ubiquitin-like protein URM1 (*24*). The isopeptide bond formed between the ubiquitin C terminus and the amine present in the lysine side chain is very stable over a range of temperatures and pH. On the other hand, the ester bond formed between the ubiquitin C terminus and the hydroxyl group present in other noncanonical targets (serine, threonine, sugars, and the bacterial liposaccharide) is hydrolyzed in mild basic conditions and at relative low temperatures. Because of the intrinsically labile nature of the ester bond and the lack of high-resolution dedicated analytical tools, the identification of ubiquitin-conjugating enzymes able to target residues other than lysine remains challenging. Here, we develop a Matrix-Assisted Laser Desorption/Ionization–Time-Of-Flight (MALDI-TOF) Mass Spectrometry (MS) approach to systematically interrogate E2s for their ability to ubiquitylate amino acids and biomolecules other than lysine. We identify a family of E2s (UBE2Qs) that ubiquitylates not only residues such as serine and threonine but also other biomolecules, including glucose and complex sugars. The UBE2Q family is distinct from canonical E2s in several aspects: They do not have the well-conserved histidine-proline-asparagine (HPN) triad that characterizes canonical E2s and have an extended N-terminus. We used protein modeling tools to generate a structural model and to predict and validate UBE2Qs activity determinants by mutational and biochemical analyses. Since E2s act upstream of E3s in the ubiquitylation cascade and can interact with multiple RING E3s (*25*), they have a larger range of substrates compared to more target-specific E3s. We therefore anticipate that the discovery of E2s with noncanonical activity will have profound and wide-ranging impacts on the ubiquitylation landscape and, consequently, toward the understanding of ubiquitin-mediated biological processes.

## RESULTS

### UBE2Qs discharge ubiquitin on hydroxyl-containing biomolecules

**Fig. 1.**
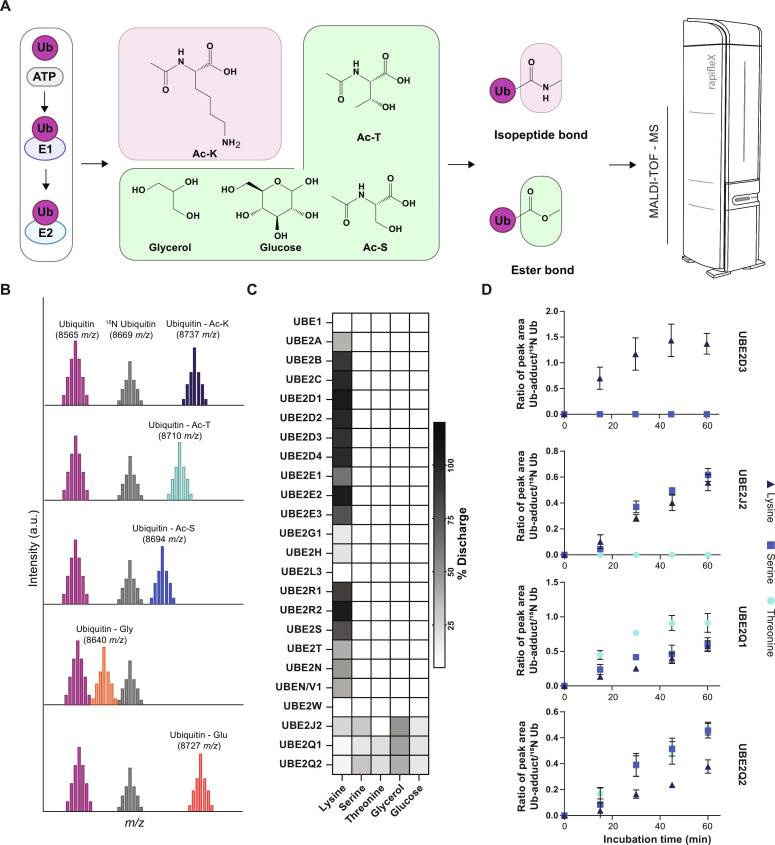
The E2 MALDI-TOF discharge assay identifies E2 noncanonical activity. Ubiquitin enzymes (E1 and E2) were incubated with adenosine triphosphate (ATP)/MgCl_2_ solution and the indicated nucleophiles. Samples were analyzed by MALDI-TOF MS (**A**). Relative quantification of E2 discharge activity on ubiquitin is obtained by use of an internal standard (^15^N Ubiquitin) (**B**). Twenty-three E2s were tested for their ability to discharge ubiquitin on the indicated nucleophiles [(**C**) heatmap data are reported as the average of at least two technical replicates]. The ability of UBE2D3, UBE2Q1, UBE2Q2, and UBE2J2 to mediate ubiquitin discharge on lysine, serine, and threonine was further validated through a time course experiment [(**D**) *N* = 3, data are shown as means ± SD from three replicates].

Although it had been determined that E3 ligases could direct ubiquitylation of noncanonical residues, it had not been determined whether E2s also direct noncanonical ubiquitylation. We therefore asked whether E2s were intrinsically reactive toward noncanonical residues. To facilitate our analysis, we developed a MALDI-TOF MS–based assay to detect the formation of ubiquitin adducts resulting from E2-conjugating discharge activity of ubiquitin on different nucleophiles (Fig. 1A), named E2 MALDI-TOF discharge assay. The E2 MALDI-TOF discharge assay relies on detection of the ubiquitin adduct formed in the presence of a nucleophile on which the E2s will discharge ubiquitin (Fig. 1, A and B). The ubiquitin adducts can be directly detected as a consequence of E2 activity over time, and absolute and relative quantification is assessed through the use of an internal standard (^15^N ubiquitin) (Fig. 1B). A panel of 23 recombinantly expressed E2s (2.5 μM final, see table S1) was tested for their ability to discharge ubiquitin onto acetyl-lysine (Ac-K), Ac-threonine (Ac-T), Ac-serine (Ac-S), glycerol, and glucose. Reactions were conducted at 30°C and incubated for 1 hour in the presence of the indicated nucleophiles (50 mM final). E2s known to work with RING-type E3s have E3-independent reactivity toward lysine (*26*). Most E2s discharged ubiquitin in the presence of Ac-K while no corresponding Ub-adduct was observed in the presence of either Ac-S, Ac-T, glycerol, or glucose (Fig. 1C). Consistent with previous literature, the HECT-specific E2-conjugating enzyme, UBE2L3, did not discharge on free lysine (*27*). In addition, UBE2W exhibited no intrinsic activity toward free lysine as previously reported (*28*, *29*) since this particular E2 specifically attaches ubiquitin to the N-terminal α-amino group of proteins (*30*). The UBE2J2-conjugating enzyme has been previously reported to be intrinsically reactive toward lysine but, unexpectedly, has also been found to be reactive toward serine (*31*). In accordance with previous studies (*31*), our data show that UBE2J2 is able to ubiquitylate glycerol, glucose, serine, and lysine but—interestingly—not threonine, indicating that a hydroxyl group alone was not sufficient to confer UBE2J2 reactivity toward its substrate. Two E2s, UBE2Q1 and UBE2Q2, were able to conjugate ubiquitin to serine, threonine, glycerol, and glucose residues but showed relatively low reactivity toward lysine residues (Fig. 1C). While both UBE2Q1 and UBE2J2 were able to ubiquitylate the more complex sugar maltoheptaose (see fig. S1, A and B), UBE2Q1 did so more efficiently than UBE2J2 (see fig.S1C). To further characterize the ability of UBE2D3, UBE2J2, UBE2Q1, and UBE2Q2 to ubiquitylate hydroxylated substrates, we tested them for discharge activity over time (Fig. 1D). UBE2D3 showed lysine-specific discharge throughout the time course experiment. UBE2Q1 showed discharge activity on all three nucleophiles but with a higher activity rate toward Ac-T compared to Ac-S and Ac-K, while UBE2Q2 showed similar reactivity toward Ac-S and Ac-T and reduced discharge on Ac-K (Fig. 1D). UBE2J2 actively discharged on both lysine and serine residues with similar rates, while it showed no discharge on threonine throughout the time course experiment.

### UBE2Q1 autoubiquitylates on nonlysine residues

**Fig. 2.**
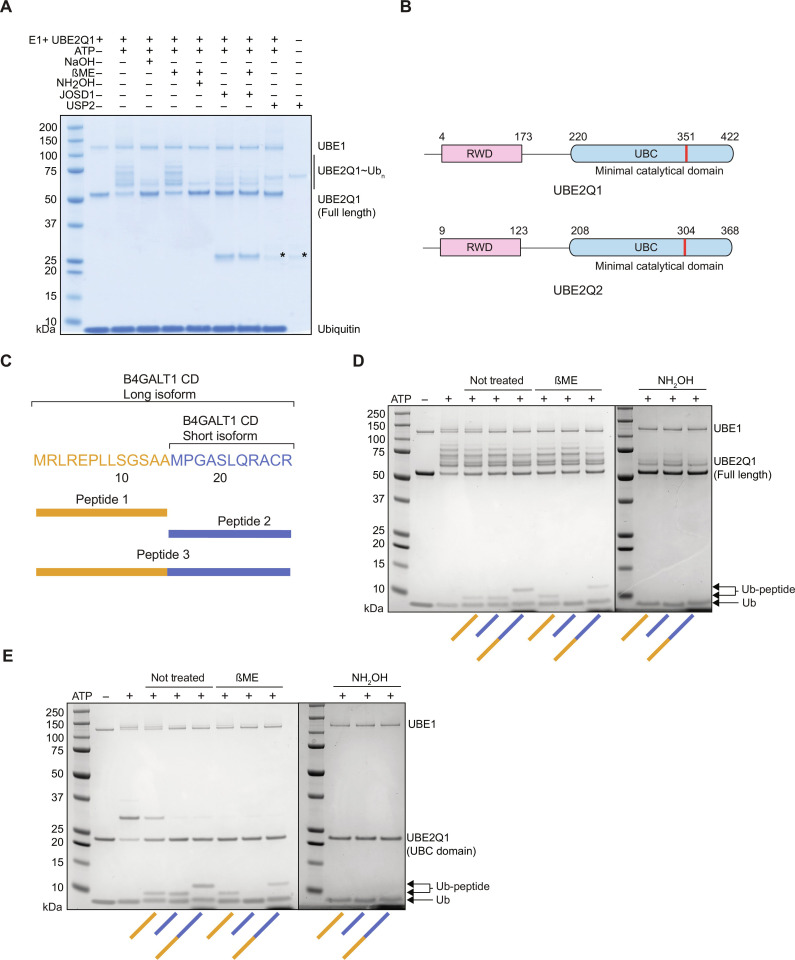
UBE2Q1 ubiquitylates B4GALT1 CD in vitro. UBE2Q1 autoubiquitylation species are sensitive to hydroxylamine (NH_2_OH) and sodium hydroxide treatment (NaOH) (**A**). Schematic of B4GALT1 CD peptides synthesized in this study (**B**). Full-length UBE2Q1 directly ubiquitylates B4GALT1 on cysteine and serine residues (**C**). UBE2Q1 and UBE2Q2 schematic representation (**D**). UBE2Q1 UBC-domain is sufficient to directly ubiquitylate B4GALT1 CD (**E**). SDS–polyacrylamide gel electrophoresis gels are representative of three independent experiments.

UBE2Q1 undergoes extensive autoubiquitylation in vitro (Fig. 2A, lane 2). To test the chemical nature of the bond linking UBE2Q1 autoubiquitylated species, the sample pH was either increased with NaOH (Fig. 2A, lane 3), treated with β-mercaptoethanol to reduce thioester bonds (Fig. 2A, lane 4), or treated with hydroxylamine to chemically cleave both ester and oxyester bonds (Fig. 2A, lane 5). The sensitivity of UBE2Q1 autoubiquitylation species to mild alkaline and hydroxylamine treatment but not thiol reduction with β-mercaptoethanol indicated that such auto-modification results from the formation of ester rather than isopeptide or thioester bond. Several UBE2Q1 autoubiquitylation species also disappeared in the presence of the deubiquitinating enzyme (DUB) JOSD1, a member of the Machado-Josephin disease DUB family previously reported to specifically cleave the ester bond linking ubiquitin to threonine substrate but unable to hydrolyze the isopeptide bond linking ubiquitin to lysine (*32*) (Fig. 2A, lane 6). JOSD1 treatment was coupled with β-mercaptoethanol reduction (Fig. 2A, lane 7), and no difference was observed compared to the JOSD1 treatment alone, further confirming that JOSD1-mediated cleavage is restricted to oxyester bond–conjugated ubiquitin. USP2, a DUB able to cleave both oxyester and isopeptide bond, removed all UBE2Q1 autoubiquitylation species.

### UBE2Q1 directly ubiquitylates B4GALT1 Cytoplasmic Domain sequence

UBE2Q1 and UBE2Q2 are characterized by an extended N terminus, which includes a protein domain shared by RING finger–containing proteins, WD-repeat-containing proteins, and yeast DEAD (DEXD)–like helicases (RWD domain) (*33*) (Fig. 2B). RWD domains have been suggested to be substrate recognition domains for ubiquitin-conjugating enzymes (*34*), but their specific function remains to be established. UBE2Q1 substrates are currently unknown; however, UBE2Q1 was found to directly interact with the cytoplasmic domain (CD) of the Golgi resident β-1,4-galactosyltransferase 1 (B4GALT1) (*35*). The B4GALT1 gene encodes three isoforms that differ in the length of their CD, a short segment that encodes 24 amino acids at the protein N termini. The three isoforms include the full-length CD or the distal (13 amino acids) or proximal (11 amino acids) segments, respectively (Fig. 2D). Notably, neither B4GALT1 shorter isoforms contain a lysine residue, but both of them have three serine residues and one cysteine residue. We hypothesized that UBE2Q1 ubiquitylates B4GALT1 CD on these noncanonical residues. Peptides belonging to the long isoform (B4GALT1 peptide 1), short isoform (B4GALT1 peptide 2), and the full-length B4GALT1 CD (B4GALT1 peptide 3) (Fig. 2D) were synthesized and incubated withan E1-activating enzyme, UBE2Q1, and adenosine triphosphate (ATP)/MgCl_2_. UBE2Q1 directly ubiquitylated both peptides 1 and 3 on a serine residue, demonstrated by the sensitivity to hydroxylamine treatment but not to β-mercaptoethanol (Fig. 2D). The ubiquitylation of peptide 2 was instead mediated by thioester bond with the cysteine, as indicated by its sensitivity to β-mercaptoethanol treatment. We speculated that the extended N terminus of UBE2Q1 might play a role in the interaction and the recognition of the B4GALT1 CD. We therefore tested a UBE2Q1 construct—containing only the UBC fold domain (UBE2Q1 UBC domain)—for its ability to directly ubiquitylate the B4GALT1 peptides. The UBE2Q1 UBC domain was still able to efficiently ubiquitylate the B4GALT1 CD on serine residues, therefore suggesting that this domain is sufficient to recognize the B4GALT1 CD sequence in vitro in the absence of its N terminus or a cognate E3 ligase (Fig. 2E). Notably, the UBE2Q1 UBC domain did not undergo extensive autoubiquitylation (Fig. 2E), suggesting that the autoubiquitylation events produced by the full-length enzyme are confined within its extended N terminus.

Intrigued by the ability of UBE2Q1 to directly ubiquitylate short lysine-free sequences, we aimed to identify additional lysine-free cytoplasmic sequences targeted by noncanonical ubiquitylation. The bone marrow stromal antigen 2 (BST2/Tetherin) stands out as an interferon-induced transmembrane protein known for impeding the release of various mammalian-enveloped viruses by tethering nascent virions to infected cell membranes (*36*). This antiviral activity of BST2/Tetherin is counteracted by the viral protein Vpu, which operates by usurping β-TrCP, a substrate adaptor for the SCF (Skp-Cullin 1-F box) E3 ubiquitin ligase complex, to mediate ubiquitylation of antiviral proteins (*37*). Notably, the short CD of BST2/Tetherin (amino acid sequence MASTSYDYCRVP; see fig. S1D) encodes two serine residues, one threonine and one cysteine residue, all identified as targets for Vpu-mediated ubiquitylation (*38*). Similar to the methodology used for B4GALT1 peptide 1, a panel of BST2/Tetherin mutant sequences was generated to discern between serine/threonine and cysteine-based ubiquitylation, facilitated by treatments with β-mercaptoethanol and hydroxylamine (fig. S1D, lanes 8 to 11 and 12 to 15). The findings revealed the efficient ubiquitylation of this short sequence by UBE2Q1, targeting both cysteine and serine residues.

In light of the observed UBE2Q1 reactivity toward cysteine residues, we tested whether UBE2Q1 could support the activity of a panel of RBR and HECT E3 ligases (HOIL-1, HOIP, Parkin, and ITCH), which have catalytic cysteines. Compared to UBE2L3, a known HECT/RBR-specific E2-conjugating enzyme, UBE2Q1, only weakly transfers ubiquitin to HOIL-1 or mediate HOIP-autoubiquitylation and formation of free ubiquitin chains (fig. S2, A and B). Similarly, UBE2Q1 does not sustain Parkin and ITCH autoubiquitylation (fig. S2, C and D). These results support the notion that UBE2Q1 is not an RBR/HECT-specific E2-conjugating enzyme.

### UBE2Q1 activity toward B4GALT1 peptide 1 depends on its closed conformation

**Fig. 3.**
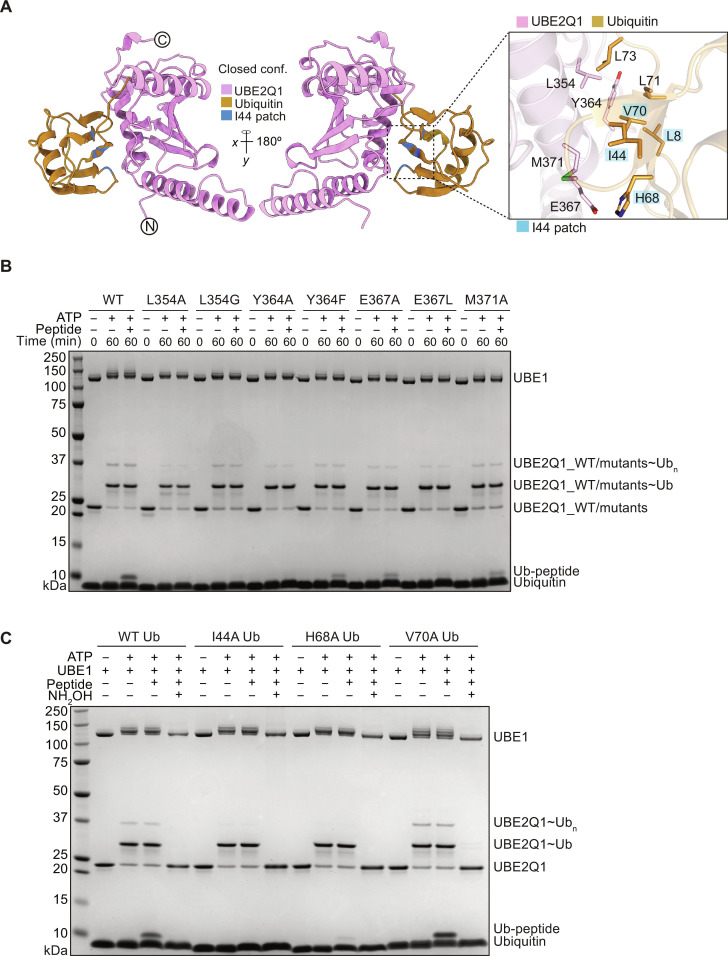
Model of UBE2Q1~Ub in closed conformation and biochemical validation. Side chains of residues at the interface between ubiquitin and UBE2Q1 highlighted in inset (**A**). UBE2Q1 residues interacting with ubiquitin were mutated and tested for discharge on B4GALT1 peptide1 (**B**). Residues of the ubiquitin Ile^44^ patch were mutated as indicated and tested for UBE2Q1-mediated discharge on B4GALT1 peptide 1 (**C**). Data are representative of three independent experiments.

In canonical ubiquitylation, RING E3s induce a folded-back or “closed” conformation of E2 with ubiquitin bound to its catalytic cysteine (*39*–*41*). This closed conformation prepares the E2~Ub complex to transfer ubiquitin onto the substrate, rendering it the “active” form. In this state, the E2 establishes an interface with the ubiquitin hydrophobic patch, centered on isoleucine-44 (the “Ile^44^ patch”) and involving Leu^8^ (L8), His^68^ (H68), and Val^70^ (V70) (*42*). To investigate the relevant conformation affecting UBE2Q1 activity in vitro, particularly in the absence of any known cognate E3 ligase, we constructed models representing both “open” and closed conformations of the UBE2Q1~Ub complex (Fig. 3A and fig. S3A). In the open conformation, specific UBE2Q1 residues, namely, Leu^405^, Ile^408^, Asn^412^, and Asp^421^, interact with the ubiquitin C terminus (fig. S3A). We systematically mutated and tested these UBE2Q1 residues for B4GALT1 peptide 1 ubiquitylation and using the E2 MALDI-TOF discharge assay (fig. S3, B and C). None of these mutants substantially reduced UBE2Q1 activity (fig. S3, B and C). We therefore moved to the closed conformation model, where UBE2Q1 Leu^354^ and Glu^367^ interact with ubiquitin Leu^73^ and His^68^, respectively. The residue Tyr^364^ on UBE2Q1 makes hydrophobic contacts with Leu^8^ and Val^70^, while Met^371^ interacts with Ile^44^. Notably, mutations in Leu^354^ and Tyr^364^ to alanine abolished UBE2Q1-mediated discharge on B4GALT1 peptide 1, while Glu^367^ to Leu and Met^371^ to Ala mutants showed markedly reduced activity (Fig. 3B). The E2 MALDI-TOF discharge assay corroborated the reduced activity of the UBE2Q1 closed conformation activity (fig. S4A) and prompted further investigation into the impact of ubiquitin mutants within the Ile^44^ patch on UBE2Q1 activity. Mutating Ile^44^ to alanine entirely eradicated UBE2Q1 activity, while mutations in Leu^8^ and His^68^ to alanine led to a substantial decrease in discharge on the B4GALT1 peptide 1 (Fig. 3C and fig. S4, B to F). The Val^70^-to-alanine mutation did not substantially affect discharge activity (Fig. 3B). These findings were consistently supported by the E2 MALDI-TOF discharge assay on nucleophiles, corroborating the observations made in the B4GALT1 peptide 1 discharge assay (see fig. S4, B to F). Collectively, these biochemical assays underscore the critical role of the closed conformation of the UBE2Q1~Ub complex in facilitating discharge on noncanonical residues.

### Tyr^343^, His^409^, and Trp^414^ are essential for UBE2Q1 noncanonical activity

Canonical E2s are characterized by a conserved and sequential histidine proline/cysteine asparagine motif (HPN motif) in the active site (*43*). Notably, the primary sequence alignment of all E2 showed that UBE2Q family, UBE2J family, and UBE2QL1 lacked the conserved HPN motif critical for the reactivity toward lysine and the formation of the isopeptide bonds (fig. S5A).

**Fig. 4.**
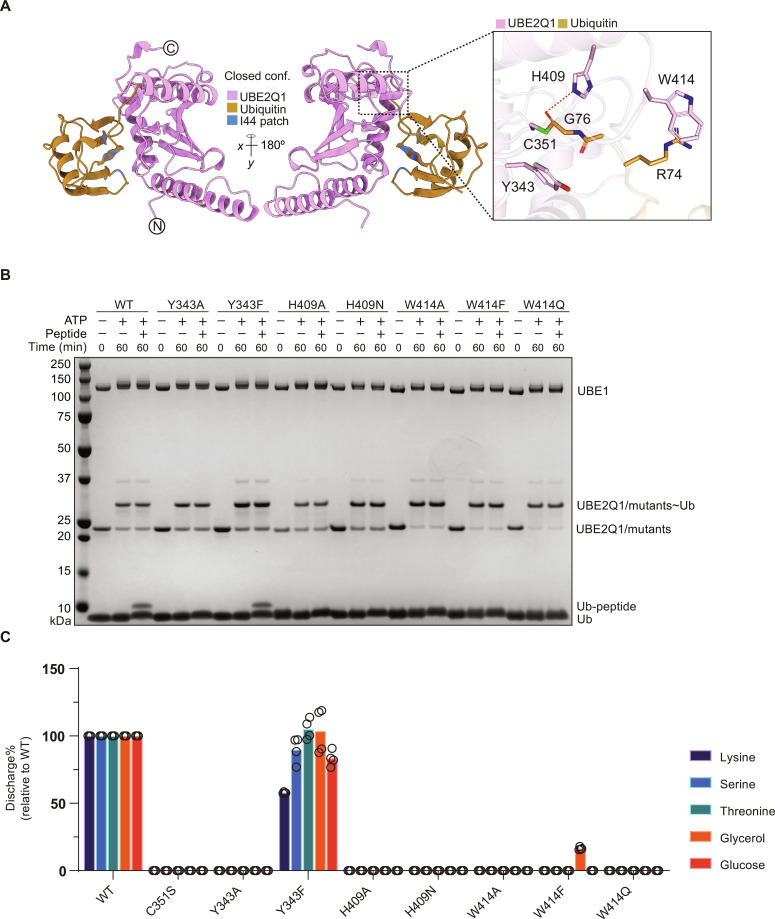
Identification of UBE2Q1 activity determinants. Model of UBE2Q1 residues Tyr^343^ (Y343), Trp^414^ (W414), and His^409^ (H409) side chains interacting with ubiquitin C terminus (**A**). Indicated UBE2Q1 mutants were tested for their ability to ubiquitylate B4GALT1 peptide 1 [(**B**) data are representative of three independent experiments] or to discharge on the indicated nucleophiles using the MALDI-TOF MS E2 discharge assay (**C**); bars represent the mean of four technical replicates.

Using MAFFT package’s L-INS-I algorithm (*44*) on JALVIEW program (*45*), we projected the primary sequence alignment of multiple metazoan species onto the UBE2Q1 3-D structure [Protein Data Bank (PDB) ID: 2QGX]. This comprehensive analysis led to the identification of three crucial residues—Tyr^343^, His^409^, and Trp^414^ (YHW triad)—in the vicinity of the catalytic cysteine (see Fig. 4A). In the closed conformation state, His^409^ engages with Gly^76^ via ionic interactions, Trp^414^ interacts with Arg^74^ through cation-Pi interactions, while Tyr^343^ forms hydrophobic interactions with Gly^76^ (Fig. 4A). Similarly, in the open conformation, His^409^ establishes ionic interactions with Gly^76^ (fig. S6A), and Tyr^343^ forms hydrophobic bonds with Gly^76^. Notably, the ubiquitin’s Arg^74^ lies within the 6.0-Å cutoff for potential cation-Pi interactions (*46*) with the Trp^414^ aromatic ring (fig. S6A).

Recognizing the potential significance of these residues in UBE2Q1 activity, we introduced alanine and conservative point mutations. All YHW triad mutants exhibited intact ubiquitin loading with thioester bond formation between E2 and ubiquitin, indicating that requisite folding was maintained to enable interaction with both the E1-activating enzyme and ubiquitin (Fig. 4B). All mutants, except for the Tyr^343^-to-phenylalanine mutation, failed to discharge on B4GALT1 peptide 1 (Fig. 4B). To further evaluate the stability of these mutants, we used a thermal shift assay: None of the YHW triad mutants displayed a significant decrease in melting temperature, suggesting that these mutations do not affect protein folding (fig. S6B).

The outcomes of the E2 MALDI-TOF discharge assay supported the findings from the B4GALT1 peptide assay (Fig. 4C). Overall, both closed and open conformation models imply the critical role of Tyr^343^, His^409^, and Trp^414^ in stabilizing the ubiquitin bound to the catalytic cysteine Cys^351^ and its subsequent discharge onto the substrate, with Tyr^343^ also contributing to the overall stability of UBE2Q1.

### UBE2Q1 prefers threonine over serine

**Fig. 5.**
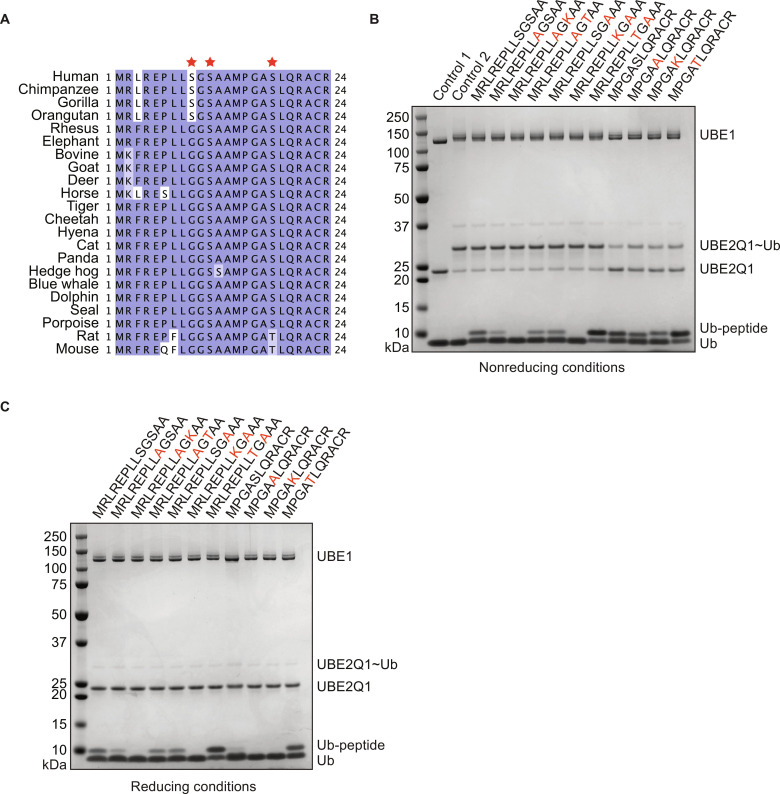
B4GALT1 CD is highly conserved. Sequence alignment of B4GALT1 CD domain in mammals (**A**). Serine residues were systematically mutated into alanine, lysine, or threonine and tested for UBE2Q1 mono-ubiquitylation in nonreducing (**B**) and reducing conditions (**C**). Data are representative of three independent experiments.

The initial MALDI-TOF E2 discharge assay time course dataset (Fig. 1D) was suggestive of an underlying preference of UBE2Q1 toward threonine rather than serine or—even more markedly—lysine. The B4GALT1 CD is highly evolutionary conserved in mammals (Fig. 5A). Serine-11 and -18 are retained or conserved mutated in all the analyzed mammalian sequences, while serine-9 is present only in primates (Fig. 5A). To test the preference of UBE2Q1 toward serine, threonine, or lysine, the two serines present within B4GALT1 peptide 1 were systematically mutated into threonine, lysine, or alanine. Substituting serine-9 in peptide 1 with alanine reduced the amount of ubiquitylation of the peptide by half, suggesting that the ubiquitylation event is distributed among serine-9 and -11. Mutating serine-9 to alanine and serine-11 to lysine completely abolished peptide ubiquitylation, further confirming an intrinsic preference of UBE2Q1 toward residues with a hydroxyl group (see Fig. 5B). UBE2Q1 showed a marked increase in ubiquitylated species when serine-11 and serine-18 were mutated into threonine but not when the S>T modification was inserted in position 9 of peptide 1 (see Fig. 5, B and C). The substitution of serine-18 into threonine in peptide 2 led to a β-mercaptoethanol–resistant ubiquitylation species (Fig. 5C), suggesting that UBE2Q1 has a strong preference for threonine even in the presence of the thiol scavenging cysteine residues. The result indicated that UBE2Q1 prefers threonine over cysteine and serine for ubiquitin discharge.

### UBE2Q1 is highly expressed in the brain

**Fig. 6.**
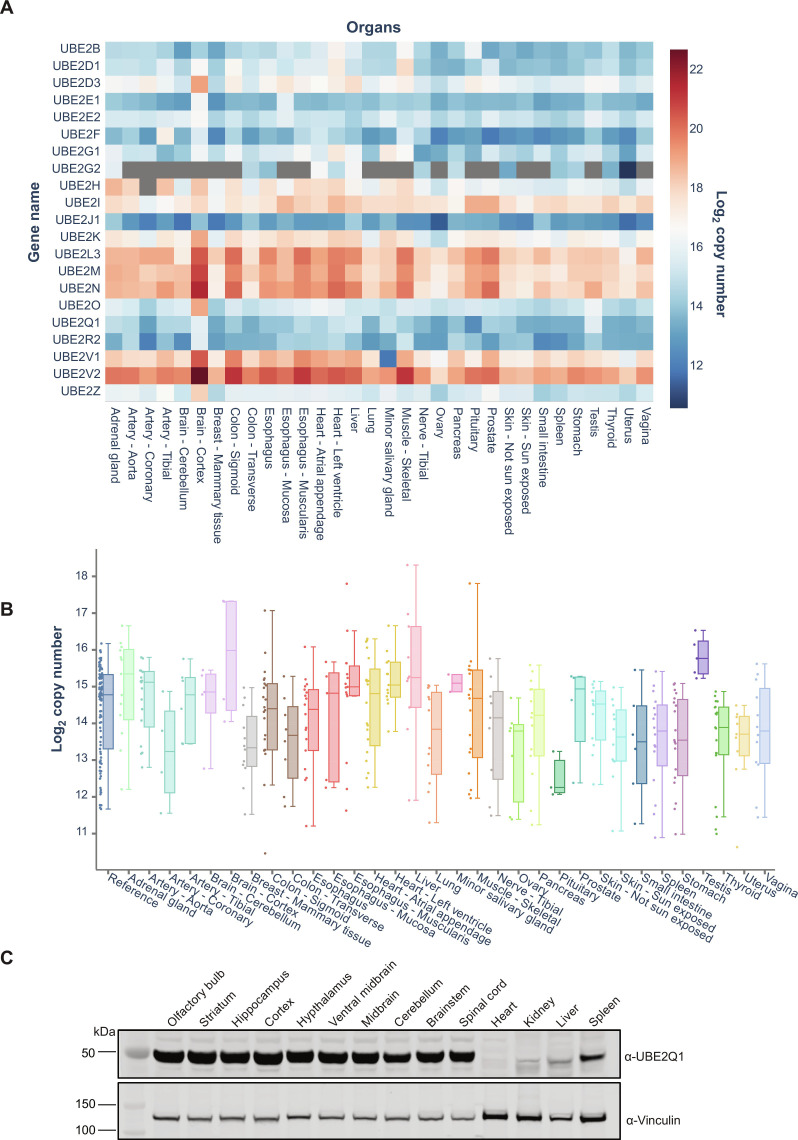
Relative expression of E2s in human and mouse tissues. E2 in vivo expression profiling obtained by data mining of publicly available quantitative proteomic dataset of 32 human tissues (**A**). Relative expression of UBE2Q1 in indicated human tissues (**B**). Western blot validation of UBE2Q1 expression in the indicated mouse tissues [(**C**) *N* = 3 biological replicates].

While the role of UBE2J2 in endoplasmic reticulum–associated protein degradation (ERAD) has been characterized, the biological role(s) of the UBE2Qs family is unclear. To determine whether UBE2Qs are tissue-specific or tissue-enriched, we interrogated a publicly available proteomic dataset in which 32 human tissues were analyzed using quantitative proteomics (*47*). Of about 40 E2s encoded in the human genome, only 21 were expressed in sufficient quantities to be detected in the dataset (Fig. 6A). UBE2Q1 and UBE2J1 were identified in all analyzed tissues, while UBE2Q2 and UBE2J2 were not detected, therefore suggesting that these E2 might be either relatively low abundant or expressed in other tissues or under specific biological conditions. UBE2Q1 was found to be relatively more expressed in brain and in testis (see Fig. 6B), suggesting a specific role in these tissues. We internally developed and validated a UBE2Q1 antibody designed for specific detection of UBE2Q1 expression across various cell lines and tissues. Antibody specificity was assessed using UBE2Q1 genetically ablated mouse embryonic stem cells (mESCs, fig. S7A). We tested the UBE2Q1 antibody against a panel of transiently overexpressed human E2s (fig. S7B) and 17 recombinantly expressed E2s (fig. S7C). The UBE2Q1 antibody was next used to confirm the relatively high UBE2Q1 expression levels in brain. Sections of mice brain and other tissue (liver, spleen, kidney, and heart) were collected from four different mice and tested for UBE2Q1 expression levels by Western blot. In agreement with MS data, UBE2Q1 was found to be highly expressed in all brain regions; a lower expression was observed in the spleen, liver, and kidney, while it was not detected in the heart (Fig. 5C).

### UBE2J2 efficiently ubiquitylates serine and cysteine residues

To date, the intrinsic ability of the Ube2J2∼Ub conjugate to react with serine or threonine has not been directly demonstrated, so neither the structural nor chemical determinants for hydroxyl attachment of ubiquitin have been identified. Both UBE2J2 and B4GALT1 are localized within the secretory pathway and are membrane bound. We therefore speculated that UBE2J2 might also ubiquitylate B4GALT1 CD.

**Fig. 7.**
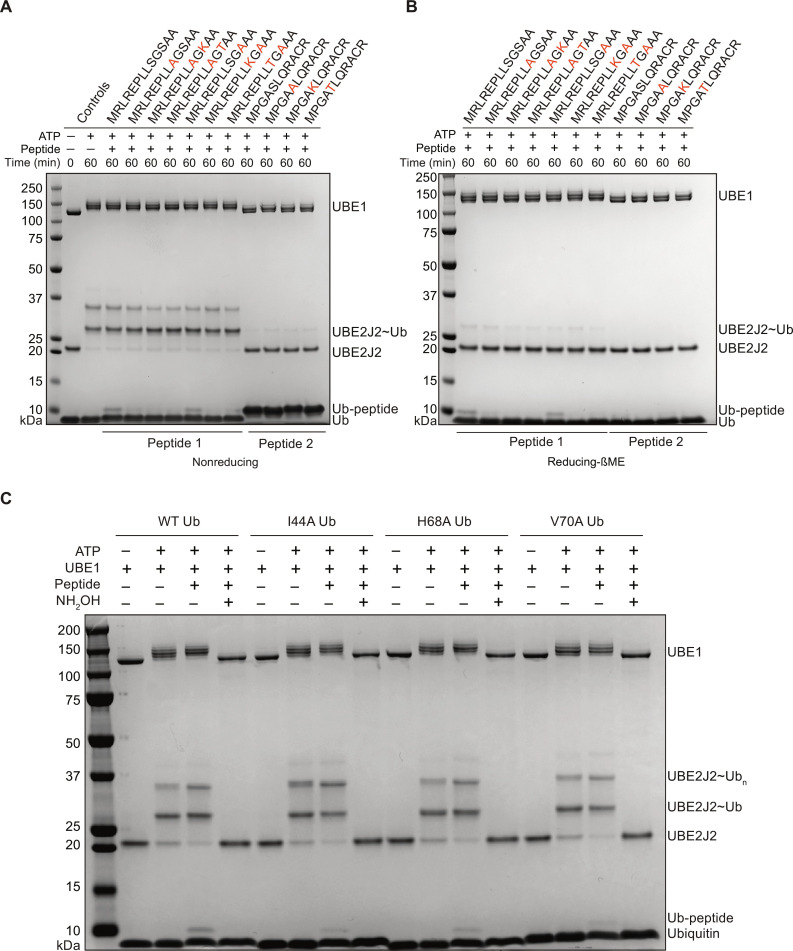
UBE2J2 ubiquitylates serine and cysteine residues. UBE2J2 facilitates the attachment of ubiquitin molecules to serine and cysteine amino acid residues within B4GALT1 peptide 1. UBE2J2-mediated ubiquitylation of B4GALT1 peptide 1 in nonreducing (**A**) and reducing (**B**) conditions. Specific mutation to WT B4GALT1 peptide 1 sequence highlighted in red [(A) and (B)]. Ile^44^ patch ubiquitin mutations do not abolish UBE2J2 activity toward B4GALT1 peptide 1 (**C**). Data are representative of three independent experiments.

UBE2J2 efficiently ubiquitylated B4GALT1 peptide 1 (Fig. 7A, lane 3); however, mutating serine on position 9 to alanine substantially reduced the discharge of the ubiquitin onto the peptide (Fig. 7A, lane 4). On the other hand, serine mutation on position 11 to alanine showed decreased ubiquitylation compared to the wild type (WT), but the discharge did not cease (Fig. 7A, lane 7). This differential effect on the pattern of the ubiquitylation highlights the specificity of the serine position on the peptide. To test the preference of serine over lysine, we mutated both serines at positions 9 and 11 to lysine within B4GALT1 peptide 1 and observed little or no ubiquitylation (Fig. 7A, lanes 5 and 8). Replacing both serines with threonine in either position did not rescue the pattern of ubiquitylation, as observed in peptide 1 (Fig. 7A, lanes 6 and 9), thus showing that serine is preferred not only over lysine but over threonine as well. Peptide 2—the cysteine containing portion of B4GALT1 CD—was strongly ubiquitylated by UBE2J2 (Fig. 7A, lanes 10 to 13). The nature of peptide 2 ubiquitylation was thioester based as demonstrated by the sensitivity of these adducts to β-mercaptoethanol treatment (Fig. 7B, lanes 10 to 13). Considering the UBE2J2 reactivity toward cysteine containing peptides, we tested whether UBE2J2 could efficiently interact with the catalytic cysteine of a panel of HECT/RBR E3 ligases: ITCH, HOIL-1, Parkin, and HOIP. UBE2J2 did support the formation of HOIL-1-Ub band and did promote the formation of free ubiquitin chains when coupled to HOIP (fig. S8, A and B). However, UBE2J2 did not interact to sustain the formation of either Parkin or ITCH autoubiquitylation species (fig. S8, C and D). Overall, these results suggest that UBE2J2 efficiently ubiquitylates serine and cysteine and productively interacts with HOIP and HOIL-1 RBR E3 ligases.

Last, we investigated the significance of closed conformation for the UBE2J2 noncanonical activity (fig. S9A). Ubiquitin I44 patch mutants were systematically tested with UBE2J2, and none of them completely halted the UBE2J2-mediated discharge on B4GALT1 peptide 1 (Fig. 7C). The results from the MALDI-TOF orthogonal testing did not perfectly mirror those obtained from the gel-based assay (fig. S9, B to E). Although some discharge on lysine persisted among the I44 patch mutants, the activity toward other nucleophiles—such as serine, glycerol, and glucose—was nearly entirely abolished by Ile^44^ and Val^70^ mutants (fig. S9, B to E). Further investigations will be necessary to comprehend the dynamics between the open and closed conformations of UBE2J2 and their direct impact on UBE2J2’s activity.

### Residues in the vicinity of catalytic cysteine are critical for UBE2J2 activity

**Fig. 8.**
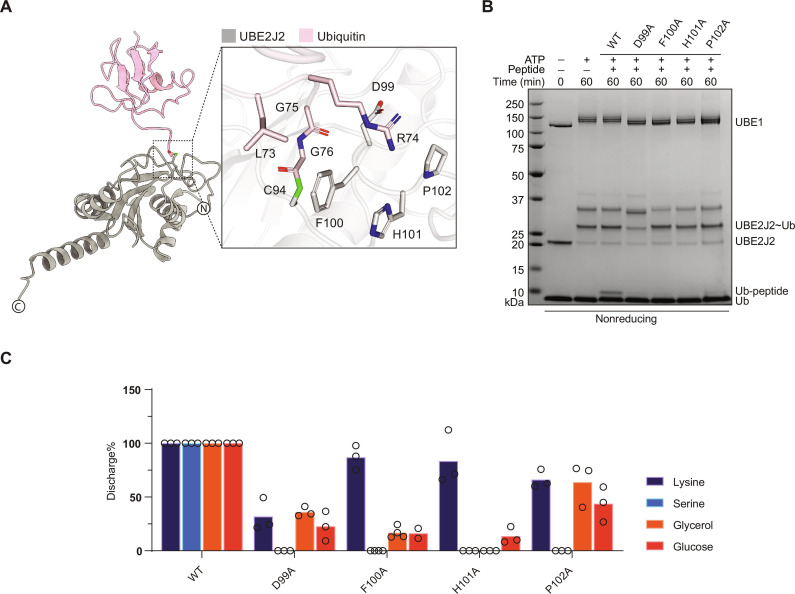
Identification of UBE2J2 activity determinants. Predicted interaction between UBE2J2 and ubiquitin in an open conformation model. Inset, side chains of residues relevant for enzymatic activity (**A**). Indicated UBE2J2 mutants were tested using B4GALT1 peptide 1 ubiquitylation [(**B**) data are representative of three independent experiments] and discharge on the indicated nucleophiles by MALDI-TOF/MS [(**C**) bars represent the mean of four technical replicates].

To identify residues relevant for UBE2J2 noncanonical activity, we built a UBE2J2~Ub model, in both open and closed conformation (see fig. S9A) using protein modeling, as for the UBE2Q1~Ub complex described above. A sequence of residues downstream of the catalytic site (Cys^94^), spanning from Leu^95^ to Thr^104^, could not be traced in the UBE2J2 apo crystal structure (PDB ID: 2F4W). This region includes two proline residues at positions 102 and 106, making it inherently flexible and unstructured. This stretch may adopt an ordered conformation upon forming a thioester bond with an incoming ubiquitin (fig. S9F). The UBE2J2~Ub open conformation model was assessed to identify the critical residues that may play a role in the discharge of ubiquitin onto the substrate. Some of the missing sequential residues Asp^99^, Tyr^100^, His^101^, and Pro^102^ from the catalytic cysteine loop in the crystal structure were found to be interacting with the C-terminal of the ubiquitin bound to the catalytic cysteine in the model (Fig. 8A, inset). To validate the UBE2J2~Ub open conformation model, we generated alanine point mutants and observed the pattern of ubiquitylation discharge onto B4GALT1 peptide 1. None of the mutants showed significant ubiquitin loading defects; however, they could no longer discharge ubiquitin onto B4GALT1 peptide 1 (Fig. 8B). We further assessed these mutations for their ability to affect the discharge onto nucleophiles by MALDI-TOF MS (Fig. 8C). The Asp^99A^ mutant showed a 50 to 70% reduction in the reactivity toward lysine, glycerol, and glucose and blocked the formation of Ub-Serine adducts. Mutating Phe^100^ and His^101^ into alanine nearly abolished discharge on the hydroxyl group of serine, glycerol, and glucose but only partially reduced the discharge on lysine. Pro^102^ to Ala showed a mixed phenotype, with about 50% reduction of the lysine-mediated discharge and a 70 and 85% reduction in the reactivity toward glycerol and glucose, respectively. Overall, these results define UBE2J2 as a hybrid E2-conjugating enzyme: Similarly to canonical E2s, UBE2J2 has sequential histidine and proline residues that are highly conserved and structurally necessary in canonical E2s although dispensable for isopeptide bond formation (*48*). However, UBE2J2 lacks the asparagine residue, previously deemed essential in the isopeptide bond catalysis (*48*) while retaining a critical aspartic acid (Asp^99^) in line with other—lysine-specific—E2s (*27*, *49*).

## DISCUSSION

E2s play an upstream role within the ubiquitylation cascade: Interacting with multiple E3 ligases has the potential to tag a wide range of substrates with ubiquitin. The HPN motif is highly conserved in canonical E2s (*43*, *50*) where it underpins key structural and functional roles in mediating ubiquitylation (*48*, *51*).

The lack of such a conserved motif defined the early discovery of the UBE2Js family, initially known as noncanonical ubiquitin–conjugating enzymes—NCUBE1 and NCUBE2. UBE2J2’s involvement in nonlysine-mediated degradation within the ERAD is well documented (*21*, *23*, *31*). Notably, the intrinsic reactivity of a specific E2 is likely predictive of its product nature (*26*). This is evident in cases like UBE2L3, whose lack of lysine reactivity accurately predicted its role as a HECT/RBR-specific E2-conjugating enzyme (*27*). Similarly, UBE2W does not ubiquitylate free lysine as its preference for N-terminal α-amino group of proteins has been demonstrated (*29*, *30*). This suggests that an E2 enzyme’s inherent reactivity will dictate the characteristics of its resulting products in vivo. UBE2J2 and UBE2Q1 were found able to directly ubiquitylate the 24–amino acid B4GALT1 and BST2/Tetherin CD on serine and/or threonine residues in the absence of an E3 ligase, under in vitro conditions. Further exploration is necessary to pinpoint UBE2Qs and UBE2Js in vivo substrates, a task complicated by the vast number of potential interacting E3 complexes and the technical challenges of detecting noncanonical ubiquitylation on a proteomic level. To date, there is no validated UBEQ1-specific E3 ligase. However, UBE2Q1 was identified as an E2-conjugating enzyme responsible for mediating the degradation of N-terminal glycine degrons, coordinated by Cullin-2 complex, which suggests that UBE2Q1 might be cognate of adaptor-like E3 ligases, specifically the Cul2^ZYG11B^ and Cul2^ZER1^. The reactivity of UBE2Q1 toward the B4GALT1 CD was negatively affected by mutation of the ubiquitin’s Ile^44^ patch. It might be possible to assume that the UBE2Q1~Ub complex dynamic is shifted toward a closed-conformation, highly reactive state to complement the activity of adaptor-like E3 ligases.

Ubiquitylation of lysine-free, short amino acid sequences is not uncommon. The cytoplasmic tail of T cell receptor α, consisting of the residues RLWSS, was previously found to be ubiquitylated by the combined action of UBE2J2 and HRD1 (*23*). UBE2J2 was also found to ubiquitylate the cytoplasmic tail of the major histocompatibility complex class I heavy chains by interacting with the γ-HV68 murine virus K3 ligase (mK3) (*31*). Similarly, the viral adaptor protein VPU mediates ubiquitylation of the cytoplasmic tail of CD4 (*52*) and the CD of BST2/Tetherin (*38*) on serine and threonine residues. All of these membrane proteins only have few residues that are accessible for ubiquitylation. It might be speculated that noncanonical E2-conjugating enzymes have evolved to target those short, lysine-free sequences. UBE2Qs and UBE2Js are not located on the same cellular compartment. UBE2J2 is bound to the membrane of the endoplasmic reticulum; by contrast, UBE2Q1 is reported as located mainly in the cytoplasm. The difference in cellular compartmentalization of these E2s might suggest that these E2s affect different ubiquitylation substrates within different cellular compartments. The high expression of UBE2Q1 in the brain correlates with its reported role in traumatic brain injury and frontotemporal dementia (*53*, *54*). Moreover, UBE2Q1 has been found to have a pleiotropic effect on fertility by playing a fundamental role during the implantation and development of embryos and subsequent pregnancy viability (*55*). UBE2Q1^−/−^ female mice show significantly reduced fertility rates. We anticipate that the discovery of UBE2Q1 noncanonical activity will help to resolve the molecular mechanisms that drive such dramatic phenotype.

Notably, a second UBE2Q1 ortholog, UB2QL1, known to be important in the clearance of damaged lysosomes (*56*), was inactive in our in vitro assay. However, it is likely to have the same noncanonical activity as UBE2Q1 and UBE2Q2. Similarly, UB2J1 was also found to be inactive in vitro. This suggests that both enzymes might require the cellular environment, specific cofactors, or specific posttranslational modifications to function. In total, 5 of about 39 ubiquitin E2 enzymes are likely to have noncanonical activity based on their relatedness to the enzymes identified here. In summary, the growing number of ubiquitin enzymes able to target amino acids other that lysine, particularly E2s, which act upstream of E3 ligases, suggest that there is a vast pool of potential substrates that might be subjected to noncanonical ubiquitin modifications. Thus, noncanonical ubiquitylation might have more far-reaching biological impacts than previously anticipated.

## MATERIALS AND METHODS

### E1-activating enzyme and E2-conjugating enzyme expression and purification

Human recombinant 6His-tagged UBE1 (DU32888) was expressed and purified from Sf21 cells using Ni-nitrilotriacetic acid (Ni-NTA) agarose and standard protocols. Human E2s were all expressed as 6His-tagged fusion proteins in BL21 cells and purified via their tags using standard protocols as previously described (*57*). Briefly, BL21(DE3) codon plus cells were transformed with the appropriate constructs (see Table 1), colonies were picked for overnight cultures, which were used to inoculate 6 liters of lysogeny broth (LB) supplemented with ampicillin (100 μg/ml) or kanamycin (50 μg/ml). The cells were grown in Infors incubators, shaking at 200 rpm until the optical density at 600 nm (OD_600_) reached 0.5 to 0.6, and then cooled to 16° to 20°C. Protein expression was induced with 250 μM isopropyl-β-d-thiogalactopyranoside (IPTG), and the cells were left further to shake overnight. The cells were collected by centrifugation at 4200 rpm for 25 min at 4°C in a Beckman J6 centrifuge. The cells were resuspended in ice-cold lysis buffer [50 mM tris-HCl (pH 7.5), 250 mM NaCl, 25 mM imidazole, 0.1 mM EGTA, 0.1 mM EDTA, 0.2% Triton X-100, leupeptin (10 μg/ml), 1 mM PefaBloc (Roche), and 1 mM dithiothreitol (DTT)] and sonicated (45% amplitude, 10-s pulse on/pulse off for a total of 60 s). Cell debris was removed by centrifugation at 18,500*g* for 45 min at 4°C. The supernatant was incubated for 60 min with Ni-NTA agarose (Expedeon) and then washed five times with 10 column volumes of the lysis buffer and then two times with 10 column volumes in 50 mM Hepes (pH 7.5), 150 mM NaCl, 0.015% Brij35, and 1 mM DTT. Protein fractions were eluted by either incubation with the lysis buffer containing 0.4 M imidazole or by overnight treatment with tobacco etch virus protease to cleave the tag. The relevant fractions were pooled, concentrated, and buffer exchanged into 50 mM Hepes (pH 7.5), 150 mM NaCl, 10% glycerol, and 1 mM DTT and stored at −80°C.

**Table 1.** Material availability.

Reagent or resource	Source	Identifier
Bacterial strains		
BL21(DE3) cells	New England Biolabs	Catalog no. C2527H
Chemicals, peptides, and recombinant proteins		
Glutathione Sepharose 4B	Expedeon	Catalog no. AGSCUST
GST-UBE2Q1_full length 1–422	MRC-PPU Reagents and Services	DU4213
GST-UBE2Q1 220–422 (end) (WT)	MRC-PPU Reagents and Services	DU23956
GST-3C-UBE2Q1 D220-G422 C351S	MRC-PPU Reagents and Services	DU61423
GST-3C-UBE2Q1 Y343A D220-G422	MRC-PPU Reagents and Services	DU73951
GST-3C-UBE2Q1 Y343F D220-G422	MRC-PPU Reagents and Services	DU77761
GST-3C-UBE2Q1 L345A D220-G422	MRC-PPU Reagents and Services	DU73272
GST-3C-UBE2Q1 L405A D220-G422	MRC-PPU Reagents and Services	DU73273
GST-3C-UBE2Q1 I408A D220-G422	MRC-PPU Reagents and Services	DU73274
GST-3C-UBE2Q1 H409A D220-G422	MRC-PPU Reagents and Services	DU73264
GST-3C-UBE2Q1 H409N D220-G422	MRC-PPU Reagents and Services	DU73955
GST-3C-UBE2Q1 N412A D220-G422	MRC-PPU Reagents and Services	DU73263
GST-3C-UBE2Q1 W414A D220-G422	MRC-PPU Reagents and Services	DU73277
GST-3C-UBE2Q1 W414F D220-G422	MRC-PPU Reagents and Services	DU73954
GST-3C-UBE2Q1 W414Q D220-G422	MRC-PPU Reagents and Services	DU73975
GST-3C-UBE2Q1 Y415A D220-G422	MRC-PPU Reagents and Services	DU73276
GST-3C-UBE2Q1 P417G D220-G422	MRC-PPU Reagents and Services	DU73945
GST-3C-UBE2Q1 P417A D220-G422	MRC-PPU Reagents and Services	DU73946
GST-3C-UBE2Q1 P418A D220-G422	MRC-PPU Reagents and Services	DU73270
GST-3C-UBE2Q1 P418G D220-G422	MRC-PPU Reagents and Services	DU73271
GST-3C-UBE2Q1 D421A D220-G422	MRC-PPU Reagents and Services	DU73275
GST-3C-UBE2Q1 D421L D220-G422	MRC-PPU Reagents and Services	DU73952
GST-3C-UBE2Q1 Y364F D220-G422	MRC-PPU Reagents and Services	DU77760
GST-3C-UBE2Q1 E367A D220-G422	MRC-PPU Reagents and Services	DU77636
GST-3C-UBE2Q1 E367L D220-G422	MRC-PPU Reagents and Services	DU77771
GST-3C-UBE2Q1 M371A D220-G422	MRC-PPU Reagents and Services	DU77635
GST-3C-UBE2Q1 L354A D220-G422	MRC-PPU Reagents and Services	DU77640
GST-3C-UBE2Q1 L354G D220-G422	MRC-PPU Reagents and Services	DU77637
GST-3C-UBE2Q1 Y364A D220-G422	MRC-PPU Reagents and Services	DU77639
GST-3C-UBE2Q1 Y364F D220-G422	MRC-PPU Reagents and Services	DU77760
GST 3C UBE2J2 T11-T183	MRC-PPU Reagents and Services	DU61848
GST 3C UBE2J2 T11-T183 D99A	MRC-PPU Reagents and Services	DU72870
GST 3C UBE2J2 T11-T183 D99N	MRC-PPU Reagents and Services	DU75386
GST 3C UBE2J2 T11-T183 D99K	MRC-PPU Reagents and Services	DU75387
GST 3C UBE2J2 T11-T183 F100A	MRC-PPU Reagents and Services	DU72894
GST 3C UBE2J2 T11-T183 H101A	MRC-PPU Reagents and Services	DU72871
GST 3C UBE2J2 T11-T183 P102A	MRC-PPU Reagents and Services	DU72872
His-TEV-UBE1	MRC-PPU Reagents and Services	DU32888
Ubiquitin-SATGSHHHHHHG	MRC-PPU Reagents and Services	DU21990
Ubiquitin (tagless)	MRC-PPU Reagents and Services	DU 20027
Ubiquitin L8A (tagless)	MRC-PPU Reagents and Services	DU71833
Ubiquitin I44A (tagless)	MRC-PPU Reagents and Services	DU75710
Ubiquitin H68A (tagless)	MRC-PPU Reagents and Services	DU71824
Ubiquitin V70A (tagless)	MRC-PPU Reagents and Services	DU75713
pET15b 6His HOIL	MRC-PPU Reagents and Services	DU32155
GST-TEV-HOIP 697-1072	MRC-PPU Reagents and Services	DU22629
6His SUMO parkin (223S)	MRC-PPU Reagents and Services	DU40847
6His 3C UBCH7	MRC-PPU Reagents and Services	DU12798
pET156P-1-JOSD1	MRC-PPU Reagents and Services	DU20956
GST-3C--USP2	MRC-PPU Reagents and Services	DU13025
Ac-Lys (nucleophile)	BACHEM	Product no. 4033018
Ac-Ser (nucleophile)	Fluorochem	Product no. 10-F505529
Ac-Cys (nucleophile)	BACHEM	Product no. 4031426
Ac-Thr (nucleophile)	Apollo Scientific	Catalog no. OR36345
B4GALT1 and BST-2/Tetherin peptides	Peptide 2.0	Custom order
**Software and algorithms**		
Prism	GraphPad, version 10.0.3	https://graphpad.com/scientific-software/prism/
PyMOL	Schrodinger	https://pymol.org/2/
Adobe Illustrator	Adobe	https://adobe.com/uk/products/illustrator.html
ChimeraX	UCSF ChimeraX	https://cgl.ucsf.edu/chimerax/
JALVIEW	University of Dundee	https://jalview.org/

### Screening of E2s activity by MALDI-TOF MS

A total of 23 recombinant E2s (see Table 1) were expressed and adjusted to a final concentration of 2 μM into a mixture containing UBE1 (200 nM), 2 mM ATP, 20 mM MgCl_2_, 2 mM tris(2-carboxyethyl)phosphine (TCEP), and 1× phosphate-buffered saline (PBS) buffer [corresponding to phosphate buffer 10 mM, 2.7 mM potassium chloride, 137 mM sodium chloride, and 1.76 mM potassium phosphate (pH 7.5)]. Five microliters of enzymatic mixture was then dispensed using an electronic 16-multichannel pipette into a Lowbind 384 Eppendorf plate. Stock solution of Ac-K, Ac-S, Ac-T, glycerol, and glucose was prepared at the final concentration of 500 mM and pH adjusted to ~7.5. Two microliters of Ac-K, c-S, Ac-T, glycerol, or glucose were independently added to the enzymatic mixture. The reaction was started by adding 5 μl of ubiquitin (2 μM) in 1× PBS. The assay plates were covered with a self-adhesive aluminum foil and incubated at 30°C for the indicated time point(s) in an Eppendorf ThermoMixer C (Eppendorf) equipped with a ThermoTop 288 and a SmartBlock polymerase chain reaction (PCR) 384. The reactions were stopped by adding 6% trifluoroacetic acid supplemented with ^15^N Ubiquitin (2 μM). Samples were spotted on a 1536 AnchorChip MALDI plate using the Mosquito nanoliter pipetting system (TTP Labtech) as previously reported (*57*–*60*). Detection by MALDI-TOF MS was also performed as previously described (*58*). Briefly, all samples were acquired on a high-resolution Rapiflex MALDI-TOF MS (Bruker Daltonics, Bremen, Germany) equipped with Compass for flexSeries 2.0, FlexControl version 4.0, Build 48, and FlexAnalysis version 4.0, Build 14. Sample spectra were collected in automatic mode using AutoXecute (Bruker Daltonics), fuzzy control parameters were switched off, initial laser power was set to “from Laser Attenuator”, and accumulation parameters were set to 4000 satisfactory shots in 500 shot steps. Movement parameters have been set to “Walk on Spot”. Spectra were processed using FlexAnalysis software and the sophisticated numerical annotation procedure peak detection algorithm, setting the signal-to-noise threshold at 5. Internal calibration was performed using the ^15^N ubiquitin peak [(M + H) + average = 8669.5]. Mass corresponding to ubiquitin [Ub initial = (M + H) + average 8565.7] and ubiquitin adducts [Ub-K = (M + H) + average 8735.7; Ub-T = (M + H) + average 8709.6 *m*/*z*; Ub-S = (M + H) + average 8695.8; Ub-glycerol = (M + H) + average 8640.5; and Ub-Glucose = (M + H) + average 8729.9] were added to the mass control list. Spectra were manually checked to ensure accuracy in calibration and peak integration. Peak areas were exported as a .csv file using FlexAnalysis software and filtered using the previously described in-house GRID script (*58*). The percentage of discharge was calculated using the following equation:

(Ub Adduct peak areaN15 Ub peak area)*[N15][Ub initial]*100%

When testing ubiquitin mutants (L8A, I44A, H68A, and V70A), ^15^N internal standard was omitted because of the overlapping of the ubiquitin mutant signal, their respective adducts, and the ^15^N ubiquitin signals. Data analysis was conducted using Microsoft Excel software, and graphical representations were generated using GraphPad.

### In vitro UBE2Q1 autoubiquitylation assay

In vitro UBE2Q1 autoubiquitylation assay was performed as previously described (*32*). Briefly, the reaction was started by combining an equal volume of ubiquitin solution (40 μM) with an enzymatic mixture consisting of UBE1 (1.0 μM), ATP (5 mM), MgCl_2_ (20 mM), UBE2Q1 full-length or minimal catalytic domain (5.0 μM) in 1× PBS buffer (pH 7.5), and 0.5 mM TCEP. The reaction was incubated at 30°C for 45 min.

Compound 1 E1 inhibitor (MLN7243) was added at a final concentration of 10 μM, and the reaction was incubated for a further 15 min at room temperature. The reaction was then subaliquoted and treated with either JOSD1 (3.0 μM), USP2 (3.0 μM), or 1× PBS buffer for 30 min at 30°C. Reactions were stopped by adding 1× final lithium dodecyl sulfate–loading buffer (4× LDS loading buffer, Thermo Fisher Scientific) with or without β-mercaptoethanol and supplemented with either 1× PBS or hydroxylamine (0.5 M) or NaOH (0.5 M).

The samples were incubated for 30 min at 37°C, and the reaction mixtures were visualized using the 4 to 12% gradient SDS–polyacrylamide gel electrophoresis (SDS-PAGE) gels. The images were captured using the ChemiDoc Imaging System (Bio-Rad).

### In vitro Β4GALT1 and BST-2/Tetherin peptide ubiquitylation assay

The in vitro Β4GALT1 and BST-2/Tetherin peptide ubiquitylation assay was performed as described above for the UBE2Q1 autoubiquitylation assay with the addition of Β4GALT1 and BST-2/Tetherin peptides at a final concentration of 1.0 mM. UBE2Q1 WT (2.5 μM; full-length or catalytic domain), 2.5 μM UBE2Q1 mutants, and 2.5 μM UBE2J2 WT and UBE2J2 mutants (see Table 1) were incubated at 30°C for 1 hour. No compound 1 E1 inhibitor (MLN7243) treatment was performed, and the reaction was stopped using 4× LDS buffer (with or without 2.0 mM β-mercaptoethanol) and further incubated with either 1× PBS or 0.5 M hydroxylamine (pH 9.0), wherever indicated, for 30 min at 37°C. The reaction mixtures were visualized using the 4 to 12% gradient SDS-PAGE gels. The images were captured using the ChemiDoc Imaging System (Bio-Rad).

### Recombinant expression of UBE2Q1 (WT and mutants) and UBE2J2 (WT and mutants)

Recombinant glutathione *S*-transferase (GST) fusion proteins were expressed in *Escherichia coli* strain BL21 (DE3) cells. The cultures were grown in 2× yeast extract tryptone medium or LB media supplemented with ampicillin (100 μg/ml) to an OD_600_ of 0.6 to 0.8 and protein expression was induced with 0.4 mM IPTG. The cultures were further shaken at 180 rpm overnight at 16°C, harvested, frozen, and stored at −80°C. Cells were resuspended in 50 mM tris-HCl (pH 7.5), 300 mM NaCl, 10% glycerol, 2 mM β-mercaptoethanol, and 1 mM 4-(2-Aminoethyl)benzenesulfonyl fluoride hydrochloride (AEBSF), and lysed by sonication. Bacterial lysates were clarified by centrifugation at 30,000*g* for 45 min and incubated with glutathione Sepharose 4B resin for 45 min on a low-speed roller at 4°C. The recombinant protein-enriched resin was washed extensively first with a 50 mM tris (pH 7.5), 500 mM NaCl, and 10 mM DTT solution and then with the buffer containing a physiological amount of salt [50 mM tris-HCl (pH 7.5), 150 mM NaCl, 10% glycerol, and 1 mM DTT]. The GST tag was cleaved overnight on column incubation with 3C protease at 4°C. The purified proteins were dialyzed into PBS buffer (pH 7.5 and 0.5 mM TCEP). Protein purity was visualized by SDS-PAGE analysis, and concentrations were determined using NanoDrop. Proteins aliquots were flash-frozen in liquid nitrogen and stored at −80°C.

### Recombinant expression of UBE2L3, JOSD1, USP2, Parkin, HOIL1, and HOIP

The constructs mentioned above were incubated and shaken under conditions similar to those for UBE2Q1 or UBE2J2, as previously described (*32*, *57*, *61*). After purification, proteins were dialyzed into 1× PBS buffer (pH 7.5 and 0.5 mM TCEP). Protein purity was visualized by SDS-PAGE analysis, and concentrations were ascertained using a NanoDrop ND-1000 UV-Vis Spectrophotometer. Aliquots of proteins were flash-frozen in liquid nitrogen and kept at −80°C.

### Construction UBE2Q1~Ub and UBE2J2~Ub complex models

The crystal structures of the minimal catalytic domain of UBE2Q1 (PDB ID: 2QGX), UBE2J2 (PDB ID: 2F4W), and ubiquitin (PDB ID: 1UBQ) were used to model the UBE2Q1-Ubiquitin and UBE2J2-Ubiquitin interaction sites in both open and closed conformations. The missing stretches in UBE2Q1 and UBE2J2 crystal structures were traced using AlphaFold (*62*). UBE2Q1 and ubiquitin structures were superposed onto the template crystal structures of UbcH5c~Ubiquitin (PDB ID: 3UGB; UBE2D3~Ub) and UbcH5b~Ubiquitin (PDB ID: 3A33; UBE2D2~Ub) for the open conformation model generation, whereas crystal structures of the BIRC7-UbcH5b~Ub complex (PDB ID: 4AUQ) and the RNF38-Ub-UbcH5b~Ub complex (PDB ID: 4V3L) were used to model the closed UBE2Q1~Ub complex.

The UBE2Q1~Ub and UBE2J2~Ub complexes were subjected to preparation by Protein Preparation Wizard in Maestro Schrödinger (*63*). During preparation, a covalent bond was formed between Cys-351 of UBE2Q1 and Gly^76^ of ubiquitin residues. Similarly, a covalent bond was formed between Cys-94 of UBE2J2 and Gly^76^ of ubiquitin. Maestro Schrödinger default settings were used for assigning bond orders, missing hydrogens were added, partial charges were assigned at pH 7.0, crystalized water molecules were removed, and heavy atoms were optimized by restrained minimization using the OPLS3e force field (*64*). The figures were made using PyMOL by Schrödinger (http://pymol.org) or Chimera X (*65*).

### Purification of ubiquitin mutants

Tagless WT ubiquitin and mutant (L8A, I44A, H68A, and V70, also see Table 1) constructs were expressed in *E. coli* strain BL21 (DE3) cells. The cultures were grown in autoinduction media supplemented with kanamycin (50 μg/ml) to an OD_600_ of 1.0 and were further allowed to shake overnight at 16°C. The cells were harvested the following morning, flash-frozen, and stored at −80°C. The resuspended cells in 1× PBS were lysed by sonication, and lysates were clarified by centrifugation at 30,000*g* for 45 min. Sodium acetate (pH 4.5) was added to a final strength of 50 mM to the lysate to precipitate bacterial proteins. The acidified lysates were incubated on ice for 6 hours, and later, the precipitated host cell proteins were pelleted (by centrifugation at 30,000*g* for 45 min) and filtered using a 0.2 μM membrane filter. The clarified lysates were passed through cation exchange chromatography, and the relevant fractions were pooled and further purified by size exclusion chromatography (buffer: 1× PBS and 0.5 mM TCEP). The purified fractions were concentrated using Amicon Ultra-15 centrifugal filter unit and flash-frozen and stored at −80°C.

### Thermal shift assay

The effect of point mutations on UBE2Q1 folding was evaluated through a thermal shift assay using SYPRO Orange Protein Gel Stain at a 2× final concentration, following standard procedures. UBE2Q1 WT and mutants were diluted to 20 μM using 1× PBS and 0.5 mM TCEP. Protein melting curves ranging from 20° to 75°C with readings taken at every 0.5°C increment were generated using a CFX384 Touch Real-Time PCR Detection System (Bio-Rad) (*66*). The determination of melting temperatures (*T*_m_) was conducted using PRISM-GraphPad software (*66*).

### Sourcing and analysis of E2s expression profiling in human tissues

Raw data from “A quantitative proteome map of the human body” (*47*) were downloaded from Proteome Xchange (PXD016999) (*67*, *68*) and searched against UniProt SwissProt Human containing isoforms (downloaded on 5 October 2021) using MaxQuant (v2.0.3.1) (*69*). MS1 intensities per channel were estimated by weighting the MS1 intensity with the TMT intensities. Protein copy numbers were estimated using the proteomics ruler plugin (*70*) in Perseus (v2.0.3.0) (*71*). Data were further analyzed and plotted using Python (v3.9.0) and the packages Pandas (v1.3.3), Numpy (v1.19.0), and Plotly (v5.8.2).

### Animals and tissue processing for immunoblotting analysis

The C57BL/6J mice were obtained from Charles River Laboratories (Kent, UK) and housed in a specific pathogen–free facility in temperature-controlled rooms at 21°C, with 45 to 65% relative humidity and 12-hour light/12-hour dark cycles with free access to food and water and regularly monitored by the School of Life Science Animal Unit Staff. All animal studies were approved by the University of Dundee Ethical Review Committee and performed under a U.K. Home Officer project license. Experiments were conducted in accordance with the Animal Scientific Procedures Act (1986) and with the Directive 2010/63/EU of the European Parliament and of the Council on the protection of animals used for scientific purposes (2010, no. 63). Six-month-old C57BL/6J mice were euthanized by cervical dislocation, and peripheral tissues were rapidly washed in ice-cold PBS buffer and snap-frozen in liquid nitrogen.

Whole brain was dissected out from the skull, rapidly washed in ice-cold PBS, and placed on an ice-cooling plate under a stereomicroscope for brain subregion microdissection. Brain subregions, such as olfactory bulbs, cortex, hippocampus, striatum, hypothalamus, thalamus, midbrain, cerebellum, brainstem, and spinal cord, were dissected and collected in a single 1.5-ml microcentrifuge tube and snap-frozen in liquid nitrogen. Tissue samples were stored at −80°C until ready for processing. All tissues were weighed and homogenized in 5× volume/mg of tissue of ice-cold lysis buffer containing: 50 mM tris-HCl (pH 7.5), 1 mM EDTA (pH 8.0), 1 mM EGTA (pH 8.0), 1% Triton X-100, 0.25 M sucrose, 1 mM sodium orthovanadate, 50 mM NaF, 10 mM sodium glycerol phosphate, 10 mM sodium pyrophosphate, 200 mM 2-chloroacetamide, phosphatase inhibitor cocktail 3 (Sigma-Aldrich), and complete protease inhibitor cocktail (Roche). Tissue homogenization was performed using a probe sonicator at 4°C (Branson Instruments), with 10% amplitude and two-cycle sonication (10 s on, 10 s off). Crude lysates were incubated at 4°C for 30 min on ice before clarification by centrifugation at 20,800*g* in an Eppendorf 5417R centrifuge for 30 min at 4°C. Supernatants were collected, and protein concentration was determined using the Bradford kit (Pierce).

### UBE2Q1 antibody validation

UBE2Q1 knock-out mESCs were produced using the genome editing technique of CRISPR-Cas (*72*). The specificity of UBE2Q1 antibody was tested versus a panel of transiently overexpressed human E2s. Briefly, about 400,000 human embryonic kidney 293 cells were transfected using 1 μg of cDNA coding for various E2 fused to hemagglutinin tag (UBE2G1-HA, UBE2L3-HA, UBE2O-HA, UBE2Q1/Q2-HA, UBE2R2-HA, and UBE2S-HA; see Table 1) using PEI MAX–polyethylenimine hydrochloride (molecular weight 40,000, Polysciences). Similarly, mESCs were transfected with 1 μg of cDNA coding green fluorescent protein fused to the murine version of UBE2Q1 or UBE2Q2 (GFP-UBE2Q1 or GFP-UBE2Q2) using Lipofectamine 2000 (Invitrogen) following the manufacturer’s instructions. Twenty-four hours after transfection, whole-cell extracts were prepared by lysing cells in 50 mM tris-HCl (pH 7.5), 1 mM EDTA, 1 mM EGTA, 1% NP-40, 0.27 M sucrose, 10 mM β-glycerol phosphate, 1 mM benzamide, aprotinin (2 μg/ml), 50 μM MG132, 50 μM PR619, and 1 mM AEBSF.

Approximately 30 μg of cell lysates was loaded on a NuPAGE 4 to 12% Bis-Tris gel (Invitrogen, USA) and transferred to nitrocellulose membranes using standard procedures. Nitrocellulose membranes were stained with Fast Green (Sigma-Aldrich, F7252, 0.0001% w/v in 0.1% acetic acid) as a total protein stain and imaged at 700 nm on the CLX infrared imaging system (LI-COR Biosciences). Membranes were destained (0.1 M NaOH, 30% methanol) for 10 min, washed with dH_2_O, and blocked for 1 hour at room temperature in 1× PBS supplemented with 0.1% Tween-20 and 5% w/v nonfat dry milk, followed by incubation with primary antibody overnight at 4°C and secondary antibodies for 1 hour at room temperature. Signals were acquired using the Odyssey infrared imaging system and analyzed using Image Studio software, except for glyceraldehyde-3-phosphate dehydrogenase (GAPDH) and GFP, where signals were detected using chemiluminescence and the Chemidoc system.

The anti-UBE2Q1 primary antibody was used at the final concentration 0.5 μg/ml in 1× PBS supplemented with 0.1% Tween-20 and 5% w/v nonfat dry milk. Anti-His (BioLegend, 65202), anti-HA (Roche), anti-GFP (Roche), anti-UBE2Q2 (Abcam, Ab154797), and anti-GAPDH (Santa Cruz Biotechnology, sc-32233) were diluted 1:1000 in 1× PBS and 0.1% Tween-20 with 5% bovine serum albumin. For secondary antibodies, horseradish peroxidase–conjugated antibody was diluted in in 1× PBS supplemented with 0.1% Tween-20 (anti-mouse 1:20,000). Secondary antibodies for the CLX infrared imaging system were diluted in 1× PBS supplemented with 0.1% Tween-20 (1:20,000) and included IRDye 600CW secondary anti-rat, IRDye 600CW secondary anti-rabbit, and IRDye 800CW anti-goat. UBE2Q1 antibody specificity was also assessed toward a panel of 17 recombinantly expressed E2s (including UBE2Q2). One hundred nanograms of His-tagged purified E2s was loaded on a NuPAGE 4 to 20% Bis-Tris gel, and Western blotting was performed as described above.
