# Supplementary material for: Discovery and characterization of noncanonical E2-conjugating enzymes
Source: Sci Adv. 2024 Mar 27;10(13):eadh0123. doi: 10.1126/sciadv.adh0123 (PMC10971424; doi:10.1126/sciadv.adh0123)
Supplement: Supplementary file 1 — Figs. S1 to S9 Table S1 [file sciadv.adh0123_sm.pdf]

Science Advances
AAAS

Supplementary Materials for

Discovery and characterization of noncanonical E2-conjugating enzymes

Syed Arif Abdul Rehman *et al.*

Corresponding author: Virginia De Cesare, v.decesare@dundee.ac.uk

Sci. Adv. **10**, eadh0123 (2024)
DOI: 10.1126/sciadv.adh0123

This PDF file includes:

Figs. S1 to S9
Table S1

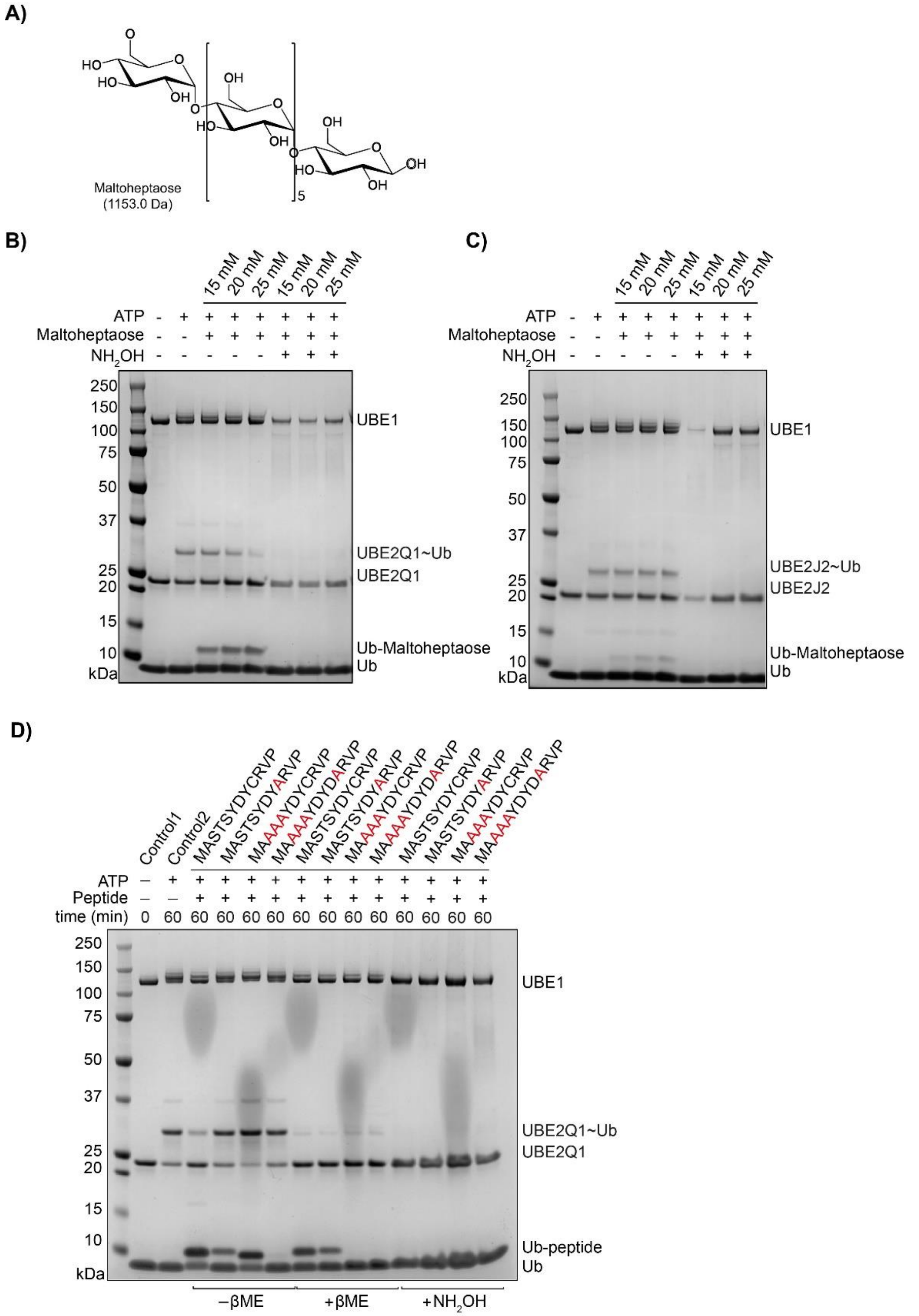

Fig. S1| UBE2Q1 and UBE2J2 ubiquitylate malthoheptaose and the cytoplasmic domain of BST-2/Tetherin. Structure of maltoheptaose (A). UBE2Q1 (B) or UBE2J2 (C) directly ubiquitylates maltohepatose in vitro. BST-2/Tetherin cytoplasmic domain sequence: Serine and Cysteine residues were systematically mutated into alanine and tested for UBE2Q1 mediated ubiquitylation. Samples were treated with β-mercaptoethanol (βME) and with hydroxylamine (NH_2OH) (D). Data representative of 3 independent experiments.

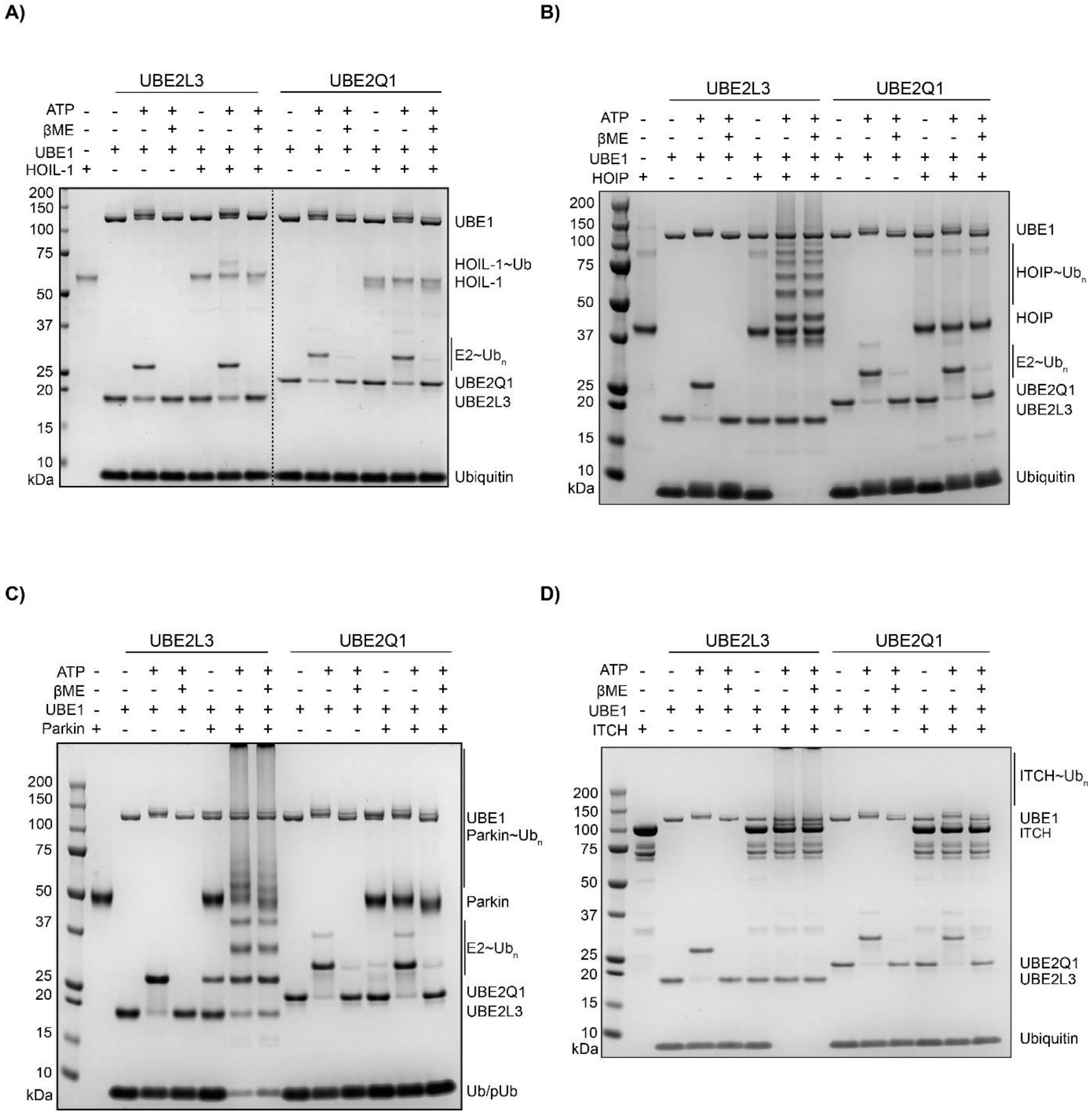

Figure S2| UBE2Q1 interaction with RBR and HECT E3 ligases. In comparison to UBE2L3, UBE2Q1 shows decreased transfer of ubiquitin to HOIL-1 (A). UBE2Q1 does not support the generation of autoubiquitylation species when coupled with HOIP (B), Parkin (C) or ITCH (D). Data representative of 3 independent experiments.

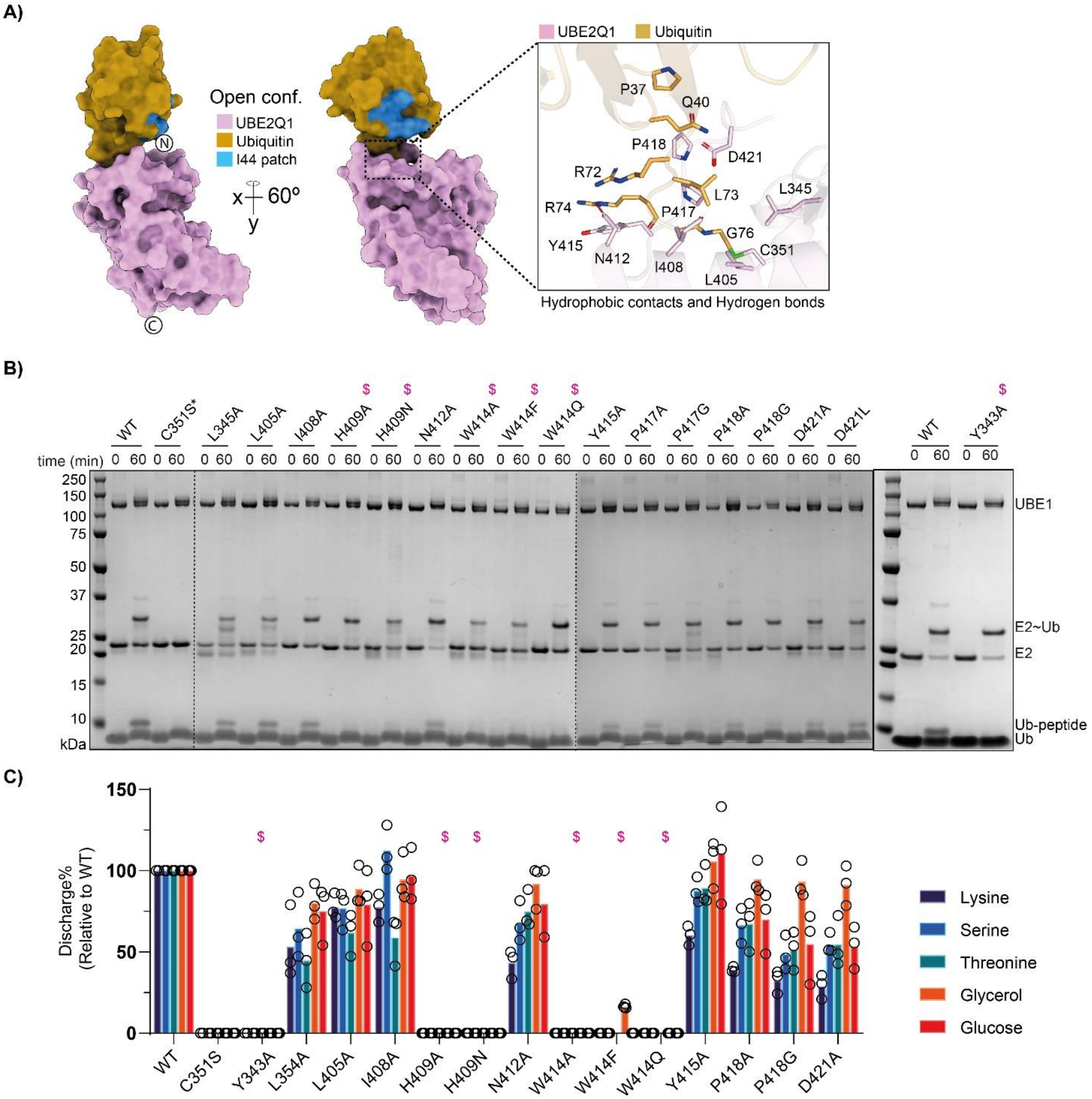

Figure S3| Model of UBE2Q1~Ub complex in open conformation. Hydrophobic contacts and hydrogen bonds between UBE2Q1 and ubiquitin are highlighted in inset (A). Indicated UBE2Q1 mutants were tested for their ability to ubiquitylate B4GALT1 peptide 1 (B), data representative of 3 independent experiments) and via MALDI-TOF discharge assay (C, bars represent mean of 4 technical replicates). $ symbolizes residues constituting YHW non-sequential triad.

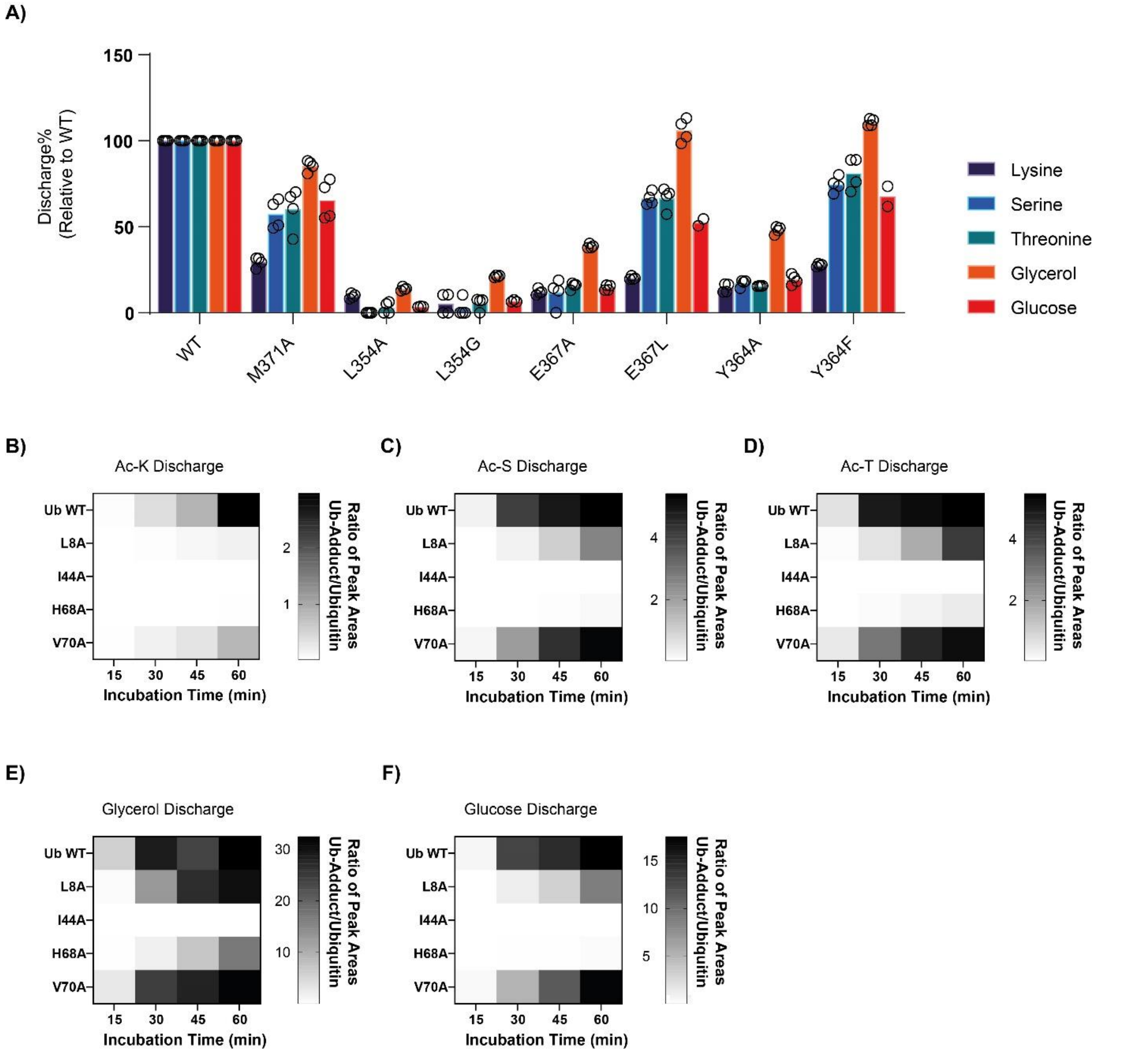

Figure S4| UBE2Q1 closed conformation and Ile44 patch ubiquitin mutants. Indicated UBE2Q1 mutants (A, bars represent mean of 4 technical replicates) and ubiquitin mutants (B-F, heatmaps represent mean of 4 technical replicates) were tested via MALDI-TOF discharge assay.

A)

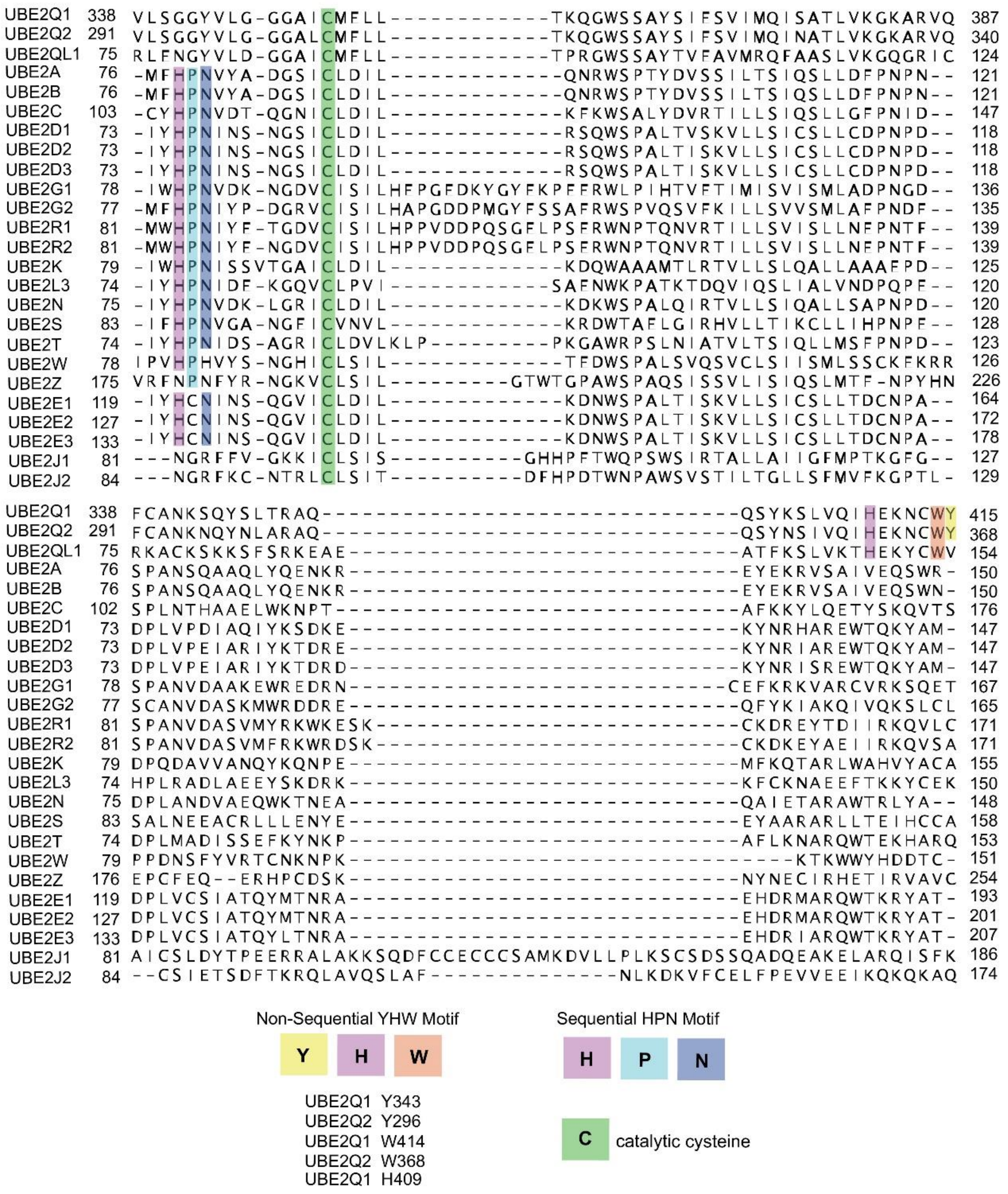

Figure S5| Alignment of the UBC domain of human E2 conjugating enzymes. Alignment of the primary sequence of 28 human E2s.reveals absence of the canonical HPN motif in the UBE2Q and UBE2J families (A). Highlighted UBE2Q1, UBE2Q2 and UBE2QL1 non-sequential YHW motif (A) and the canonical HPN triad. Catalytic cysteine marked in green.

A)

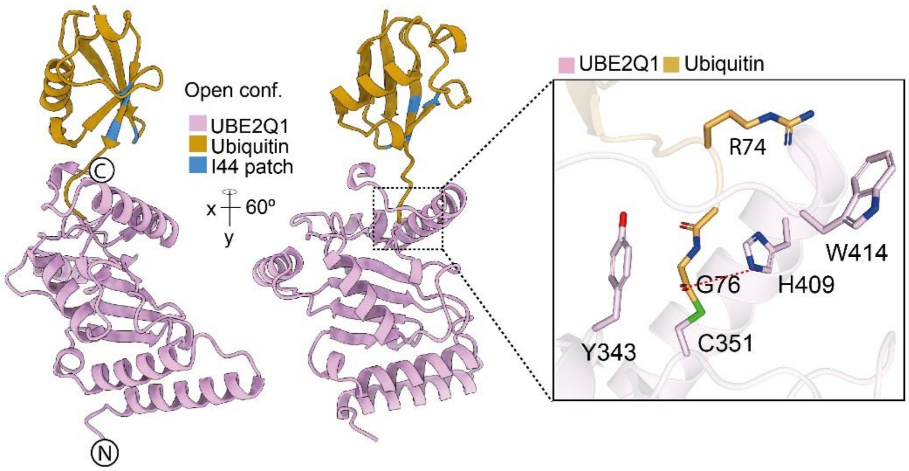

B)

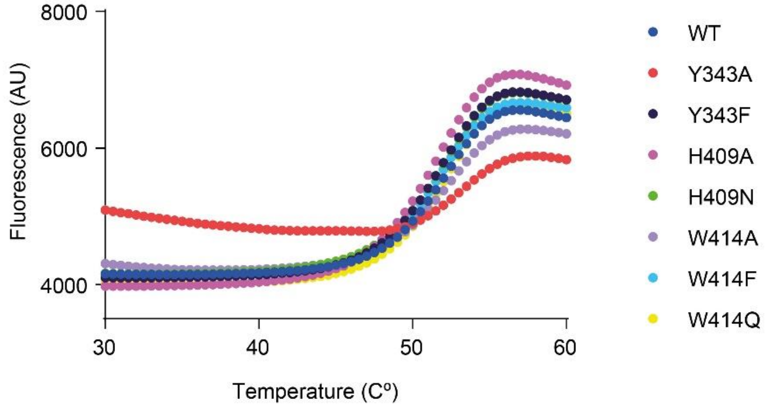

UBE2Q1	WT	Y343A	Y343F	H409A	H409N	W414A	W414F	W414Q
Tm(º)	50.81 ± 0.13	52.89 ± 0.29	50.49 ± 0.14	50.07 ± 0.16	50.57 ± 0.14	51.10 ± 0.14	50.56 ± 0.13	50.85 ± 0.15

Figure S6| YHW triad in the UBE2Q1-Ub open conformation model. Highlighted in inset side chains of Tyr343, His 409 and Trp414 (A). Thermal shift assay of YHW mutants (B, dots represent mean of 3 technical replicates)

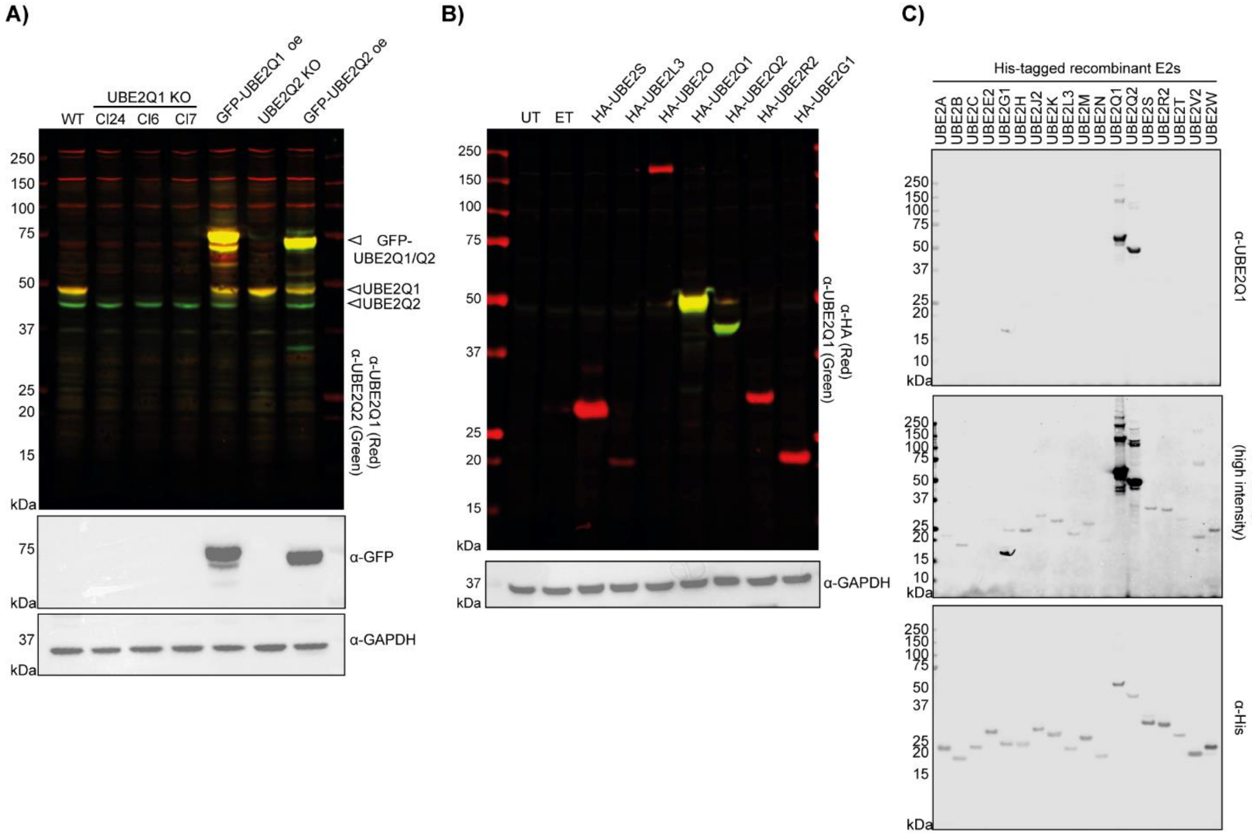

Figure S7| UBE2Q1 antibody validation. UBE2Q1 was tested for specificity against its target using WT and UBE2Q1-KO mESC cell lines transiently overexpressing GFP-UBE2Q1 or GFP-UBE2Q2 (A). Cross reactivity with other E2 conjugating enzymes was assessed by transiently over-expressing a panel of HA-tagged human E2s (B) and using recombinantly expressed E2s (C).

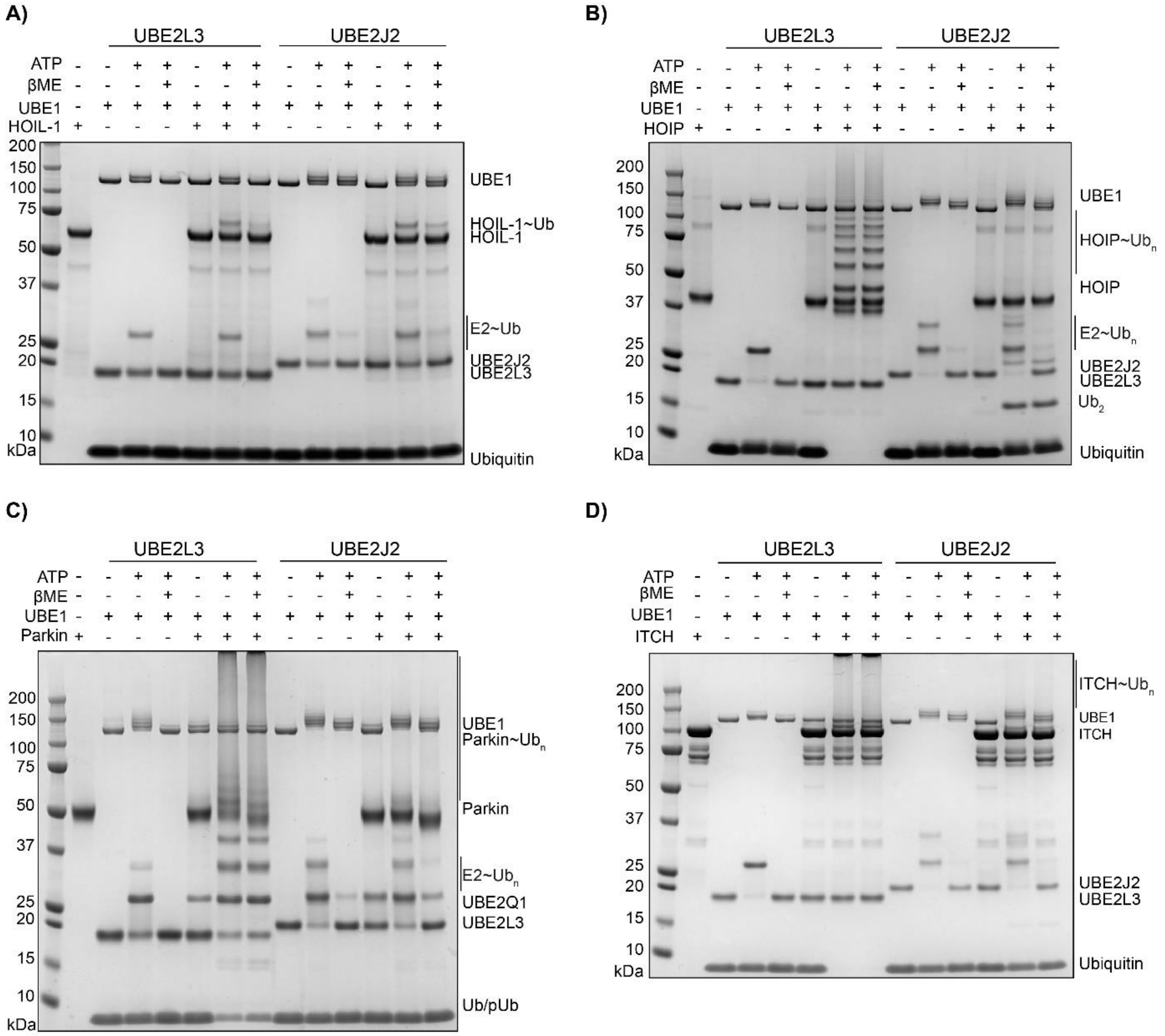

Figure S8| UBE2J2 interaction with RBR and HECT E3 ligases. UBE2J2 transfers ubiquitin to HOIL-1 and mediates free ubiquitin chains formation when combined with HOIP (A-B) UBE2J2 does not sustain the formation of Parkin and ITCH autoubiquitylation species (C-D). Data representative of 3 independent experiments

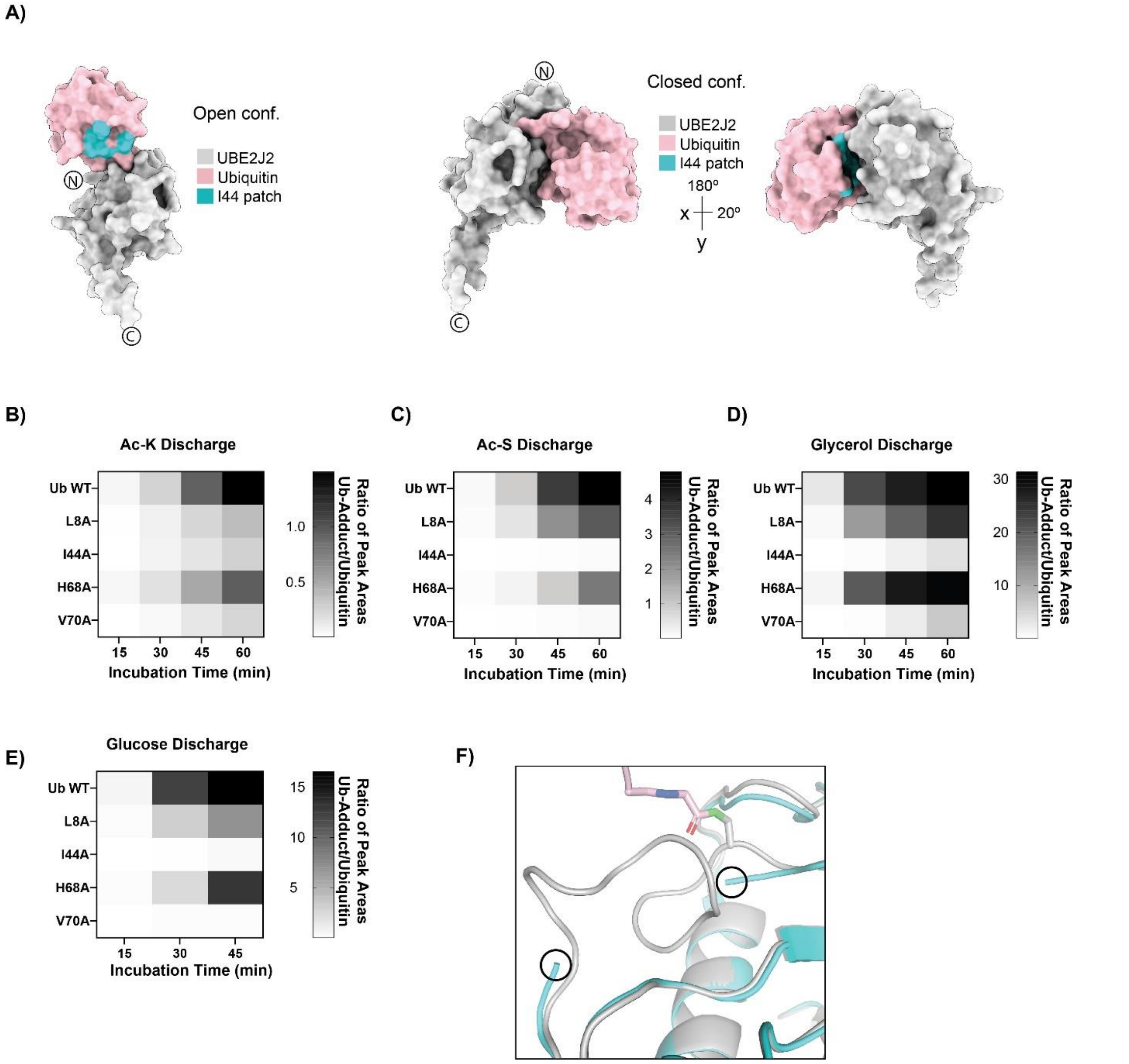

Figure S9| UBE2J2~Ub open and closed-conformation models. Protein modelling was used to predict UBE2J2~Ub open and closed conformation states (A). Indicated ubiquitin mutants were tested for their ability to support UBE2J2-mediated discharge using the MALDI-TOF discharge assay (B-E, heatmaps represent mean of 4 technical replicates). Structural alignment of the UBE2J2 apo crystal structure (shown in cyan) superposed onto the predicted model of the UBE2J2~Ub complex (depicted in grey) encompassing the highly mobile loop (spanning from Leu95 to Thr104, demarcated by black circles) that is absent in the crystal structure. The loop stabilises on interaction with the bound ubiquitin (coloured in pink) (F).

		Name	Uniprot Accession Number	Tag	Domain	Host/Source
E2 Conjugating Enzyme	1	UBE2A	P49459	His	2-152	bacteria
	2	UBE2B	P63146	His	full length	bacteria
	3	UBE2C	O00762	-	full length	bacteria
	4	UBE2D1	P51668	-	full length	bacteria
	5	UBE2D2	P62837	His	2-147	bacteria
	6	UBE2D3	P61077	His	2-147	bacteria
	7	UBE2D4	Q9Y2X8	-	full length	bacteria
	8	UBE2E1	P51965	His	full length	bacteria
	9	UBE2E2	Q96LR5	His	full length	bacteria
	10	UBE2E3	Q969T4	His	full length	bacteria
	11	UBE2G1	P62253	His	full length	bacteria
	12	UBE2H	P62256	His	full length	bacteria
	13	UBE2L3	P68036	-	full length	bacteria
	14	UBE2R1	P49427	His	2 – 236	bacteria
	15	UBE2R2	Q712K3	-	full length	bacteria
	16	UBE2S	Q16763	His	full length	bacteria
	17	UBE2T	Q9NPD8	His	full length	bacteria
	18	UBE2N	P61088	His	full length	bacteria
	19	UBE2V1	Q13404	His	full length	bacteria
	20	UBE2W	Q96B02	His	full length	bacteria
	21	UBE2Q1	Q7Z7E8	His	full length	bacteria
	22	UBE2Q2	Q8WVN8	His	full length	bacteria
	23	UBE2J2	Q8N2K1	-	full length	bacteria

Table S1| Human recombinantly expressed E2 conjugating enzymes in use in this study.
